# Transcriptional epigenetic regulation of *Fkbp1*/*Pax9* genes is associated with impaired sensitivity to platinum treatment in ovarian cancer

**DOI:** 10.1186/s13148-021-01149-8

**Published:** 2021-08-28

**Authors:** Javier Andrés Soto, Carlos Rodríguez-Antolín, Olga Vera, Olga Pernía, Isabel Esteban-Rodríguez, Maria Dolores Diestro, Javier Benitez, Fátima Sánchez-Cabo, Rafael Alvarez, Javier De Castro, Inmaculada Ibanez de Cáceres

**Affiliations:** 1 Universidad de Santander, School of Medical and Health Sciences, Masira Research Institute, Bucaramanga, Colombia; 2 Cancer Epigenetics Laboratory, INGEMM, La Paz University Hospital, Paseo de la Castellana 261, 28046 Madrid, Spain; 3 Biomarkers and Experimental Therapeutics in Cancer, Calle de Pedro Rico, 6, 28029 IdiPAZMadrid, Spain; 4 Department of Pathology, La Paz University Hospital, Paseo de la Castellana 261, 28046 Madrid, Spain; 5 Gynecologic Oncology Unit, La Paz University Hospital-IdiPAZ, Paseo de la Castellana 261, 28046 Madrid, Spain; 6 Human Genetics Group, Spanish National Cancer Research Center (CNIO), Calle de Melchor Fernández Almagro, 3, 28029 Madrid, Spain; 7 Spanish Network On Rare Diseases (CIBERER), Av. Monforte de Lemos, 3-5. Pabellón 11. Planta 0, 28029 Madrid, Spain; 8 Spanish National Center for Cardiovascular Research Center (CNIC), Calle de Melchor Fernández Almagro, 3, 28029 Madrid, Spain; 9 Hospital Universitario HM Sanchinarro, Calle de Oña, 10, 28050 Sanchinarro, Madrid, Spain

**Keywords:** *PAX9*, *FKBP1B*, Ovarian cancer, Methylation, Predictive, Platinum, Therapy

## Abstract

**Background:**

In an effort to contribute to overcoming the platinum resistance exhibited by most solid tumors, we performed an array of epigenetic approaches, integrating next-generation methodologies and public clinical data to identify new potential epi-biomarkers in ovarian cancer, which is considered the most devastating of gynecological malignancies.

**Methods:**

We cross-analyzed data from methylome assessments and restoration of gene expression through microarray expression in a panel of four paired cisplatin-sensitive/cisplatin-resistant ovarian cancer cell lines, along with publicly available clinical data from selected individuals representing the state of chemoresistance. We validated the methylation state and expression levels of candidate genes in each cellular phenotype through Sanger sequencing and reverse transcription polymerase chain reaction, respectively. We tested the biological role of selected targets using an ectopic expression plasmid assay in the sensitive/resistant tumor cell lines, assessing the cell viability in the transfected groups. Epigenetic features were also assessed in 189 primary samples obtained from ovarian tumors and controls.

**Results:**

We identified *PAX9* and *FKBP1B* as potential candidate genes, which exhibited epigenetic patterns of expression regulation in the experimental approach. Re-establishment of *FKBP1B* expression in the resistant OVCAR3 phenotype in which this gene is hypermethylated and inhibited allowed it to achieve a degree of platinum sensitivity similar to the sensitive phenotype. The evaluation of these genes at a translational level revealed that *PAX9* hypermethylation leads to a poorer prognosis in terms of overall survival. We also set a precedent for establishing a common epigenetic signature in which the validation of a single candidate, *MEST,* proved the accuracy of our computational pipelines.

**Conclusions:**

Epigenetic regulation of *PAX9* and *FKBP1B* genes shows that methylation in non-promoter areas has the potential to control gene expression and thus biological consequences, such as the loss of platinum sensitivity. At the translational level, *PAX9* behaves as a predictor of chemotherapy response to platinum in patients with ovarian cancer. This study revealed the importance of the transcript-specific study of each gene under potential epigenetic regulation, which would favor the identification of new markers capable of predicting each patient’s progression and therapeutic response.

**Supplementary Information:**

The online version contains supplementary material available at 10.1186/s13148-021-01149-8.

## Background

Ovarian epithelial carcinoma (OEC) is the most deadly of gynecological neoplasms among pelvic cancers worldwide [1]. The discouragingly high mortality rate reported for OEC is due to three key factors, the first of which is associated with the unsuccessful efforts to identify women with early stage disease, given that 75% of these patients are diagnosed at stage III-IV of the International Federation of Gynecology and Obstetrics (FIGO) staging system [2]. Unlike many other cancers, OEC has no natural barrier to prevent the widespread dissemination of tumor cells to surrounding pelvic organs and therefore it grows rapidly, metastasizes early and has a highly aggressive course [3]. The second factor involves the scarcity of biomarkers for identifying OEC and their limited transfer to clinical practice. Gene expression-based tools designed to identify postoperative and postchemotherapy predictors are only available for certain tumor types. The MammaPrint test involves the expression analysis of 70 genes to predict the probability of metastasis in breast cancer [4]. The Oncotype assay predicts recurrence after treatment in colon [5], breast [6] and prostate [7] cancer through the expression analysis of 21 genes. Several gene signature approaches associated with overall survival (OS) and therapy response in OEC have been described [8–10], but none is currently in clinical use. The third factor is treatment failure, mainly caused by resistance to conventional chemotherapy, which is evidenced by the high recurrence rates. Preliminary chemotherapy response rates (taxane–platinum) are 60–75% [11]; however, 30–40% of these patients relapse within 12 months [12, 13].

One scenario related to drug resistance involves specific cellular mechanisms [14] that influence the chemotherapy response by affecting intracellular active drug concentrations, drug–target interactions and the apoptotic effector machinery. These cancer cell-specific issues are associated with acquired somatic mutations and epigenetic changes. The assessment of DNA methylation status is the most widely used strategy for determining chemoresistance in the epigenetic context. Methylation of the proapoptotic gene *hMLH1* has been associated with acquired chemoresistance and plays a key role in this event. Patients with OEC exhibit an increase in *hMLH1* methylation after 4 cycles of platinum-based chemotherapy and when the disease reappears [15]. This characteristic has been identified in 25% of patients at the time of recurrence and has been detected in circulating tumor DNA, showing a direct relation with low OS [16]. Demethylation treatment of *RASSF1A*, *HOX10* and *HOX11* genes favors the response to carboplatin in patients with recurrence, showing a positive correlation between activity restoration and long-term disease-free periods [17]. This study therefore aimed to identify new chemoresistant epigenetic biomarkers by assessing methylome and transcriptome in cancer cell lines and cohorts of patients with ovarian cancer, supported by next-generation sequencing techniques, in search of an effective stratification that would predict each patient’s progression and therapeutic response.

## Results

### Establishment of cisplatin-resistant ovarian human cancer cell lines

**Fig. 1:**
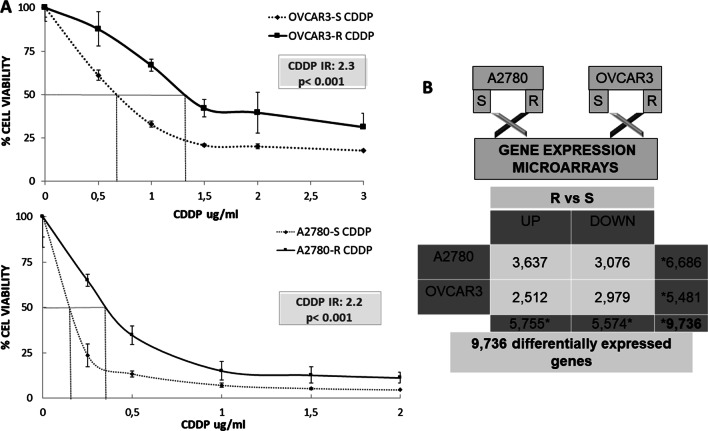
Development of resistant ovarian cancer cell lines and differential gene expression. **A** Viability curves showing the acquired resistance of A2780 and OVCAR3 cell lines. S/R CDDP identifies ovarian cancer cell lines sensitive or resistant to cisplatin, also known as *cis*-diammineplatinum (II) dichloride (CDDP). Data were normalized with respect to the untreated control (100% survival) and are shown as the mean and standard deviation of at least three independent experiments. The resistance index (RI) was calculated using the half maximal inhibitory concentration (IC50) as follows: IC50-resistant/IC50-sensitive cell line. *p* values < 0.001 indicated a significant change in drug sensitivity (Student’s *t* test). **B** Number of genes differentially expressed for each cell line in the resistance vs. sensitivity cross-analysis (adjusted *p* value ≤ 0.05) are shown. The black bar represents gene overexpression in resistance (UP), and the white bar represents gene underexpression in resistance (DOWN). We identified 3637 and 2512 overexpressed genes for A2780-R and OVCAR3-R, respectively, and 3076 and 2976 inhibited genes for A2780-R for OVCAR3-R, respectively. The overall analysis by cell type identified 6686 genes differentially expressed in A2780 and 5481 in OVCAR3. *The sum of each analysis does not have a mathematical equivalence because the probes that hybridized in duplicate for the same gene were eliminated

To identify the potential epigenetic biomarkers, we established an in vitro resistance model. OVCAR3-R and A2780-R cisplatin-resistant variants were selected after a final exposure to 0.05 and 0.5 μg/mL of cis-diammineplatinum (II) dichloride (CDDP), respectively, showing a twofold greater resistance than their corresponding parental mates (resistance index [RI] of 2.3 and 2.2, respectively; *p* < 0.001) (Fig. 1A).

### Differential gene expression between tumor phenotypes as an effect of platinum treatment

Using the statistical package limma, we identified a large number of differentially expressed genes in the ovarian cancer cell lines OVCAR3 and A2780, all of them with an adjusted p-value and false discovery rate (FDR) < 0.05. The output data contrasts of the gene expression microarrays following the previously described steps [18] helped determine the number of overexpressed or inhibited genes according to the pharmacological response and cell line. These analyses identified 9736 differentially expressed genes in the two tumor lines of ovarian cancer (Fig. 1B).

### Identification of differentially methylated candidate genes

We assessed the methylome of OVCAR-3 S/R and A2780 S/R cancer cell lines through whole genome bisulfite sequencing (WGBS), seeking to identify the genes with differential methylation among these cell phenotypes. We analyzed each tumor line’s methylation profile after filtering the CpG positions with coverage greater than 5X. We first validated *SPHK1, DCBLD2* and *CDKN2* genes by bisulfite sequencing. These genes were selected according to the methylation status assigned to each based on a differential methylation screening supported by the WGBS analysis through *β* values ​​between R > 0.7 and S < 0.3, as described in previous studies [19]. This initial test aimed to validate the selected *β* values and corroborate their exact role as a candidate selection parameter. *SPHK1* exhibited similar DNA methylation levels in the sensitive and resistant cells, while *DCBLD* and *CDKND2* showed no degree of methylation in either the sensitive or resistant phenotype (data not shown). Based on these findings, we decided to readjust the set of *β* values, adding a level of 0.2 in S and a difference greater than 0.4 in R, aiming to avoid methylated positions in R that had basal methylation already in S. However, this adjustment involved lowering the value in R to prevent losing candidates whose methylation was moderate in resistance. An analysis under the rearranged criteria identified 298,152 and 178,113 differentially methylated CpGs positions in A2780-R Vs A2780-S and OVCAR3-R Vs OVCAR3-S, respectively, encompassing 4097 genes. The interrogation of differentially methylated regions was focused on three types of genomic areas called alpha, beta and gamma. We catalogued as alpha regions those typical promoter areas near the transcription start site. Beta regions were located 2 kb and 4 kb 5’ upstream from the start of CpG islands, also known as shore and shelf regions, respectively. Gamma areas were those located within the gene body.

### Pipeline of combined approaches to identify potential predictive platinum-resistant epi-biomarkers

Our workflow started by combining suitable patient information extracted from The Cancer Genome Atlas (TCGA) with the differential expression profiles resulting from the experimental model. The implementation of the TCGA database helped establish a parallelism with findings from the in vitro analysis and thereby enabled the identification of candidates supported by clinical data. Based on information related to tumor type, treatment and clinical follow-up, we identified 576 patients, 238 of whom met the criteria for inclusion in the analysis. These criteria were having information on the platinum therapy, death within 1100 days along with gene expression data for controls and tumor samples. Of these 238 patients, 123 died within the established period and were identified as the patients with the poorest therapeutic response.

**Fig. 2:**
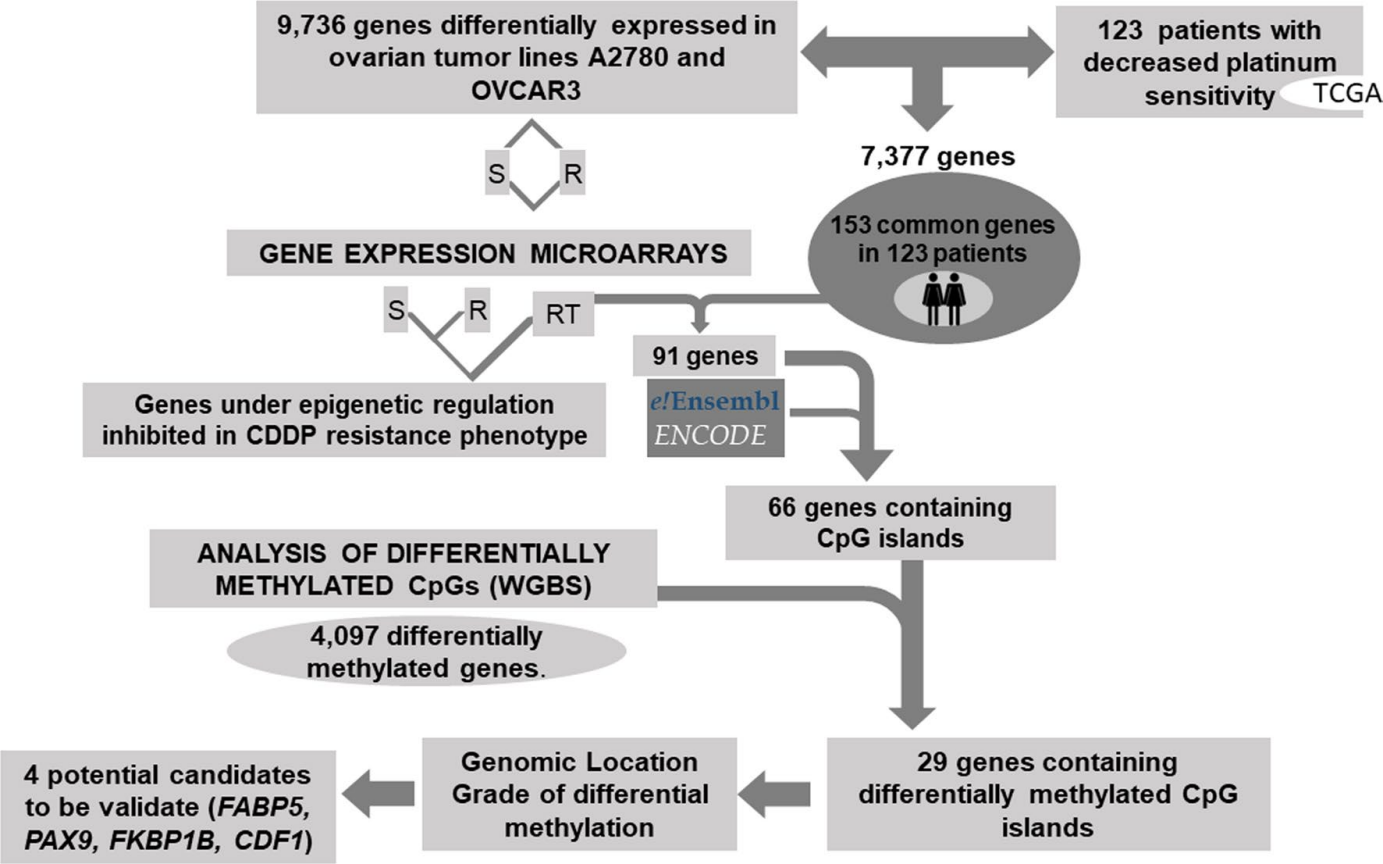
Selection of candidate target genes expressed under epigenetic regulation. Pipeline indicating the criteria followed for the selection of candidates: 1. Identification of the genes differentially expressed in the four paired cisplatin-sensitive and cisplatin-resistant cell lines A2780S/R and OVCAR3S/R that were consistent with those identified in the 123 patients interrogated in the TCGA with the same pathology and methodology (expression arrays). 2. We subsequently observed which genes coincided with those identified in the cell lines subjected to epigenetic reactivation treatment and that contained CpGs islands in the promoter regions. 3. From these, we selected those represented among the genes identified as differentially methylated in the global epigenome analysis (WGBS) conducted between the cisplatin-sensitive and cisplatin-resistant lines (4097 genes). These 29 candidates showed promoter hypermethylation as a consequence of cisplatin treatment and might therefore be involved in the onset of resistance in our in vitro model. These candidates were therefore selected by our pipeline for further screening based on the genomic location of methylated areas and the degree of methylation exhibited. This analysis was supported by external resources such as the Ensembl API, the Encyclopedia of DNA Elements (ENCODE) and patient biological data stored in TCGA

Starting from this preliminary cross-analysis, we found 7377 common differentially expressed genes between the ovarian tumor lines and the TCGA patients, 153 of which were present in all 123 selected patients (Fig. 2). To identify candidates under epigenetic regulation, this group of genes was compared with those identified by the expression microarray interrogation performed after the epigenetic reactivation treatment (RT) induced in the same cell lines. From the 153 genes, 91 were identified as re-expressed genes after drug treatment, and 66 contained CpGs islands in the promoter regions (Fig. 2). Lastly, we observed that a high percentage (44%) of these genes (29 out of 66) were represented among the genes identified as differentially methylated in the WGBS conducted between the cisplatin-sensitive and cisplatin-resistant lines (4097). These candidates showed promoter hypermethylation as a consequence of the cisplatin treatment and might therefore be involved in the onset of resistance in our in vitro model. These 29 genes were selected by our pipeline for further screening based on the genomic location of the methylated areas and the degree of methylation exhibited, leading to the selection of 4 genes for further validation (Fig. 2).

### *PAX9* and *FKBP1B* are differentially methylated in cisplatin-resistant ovarian cancer cells.

The criterion followed for the gene selection was based on the location of differentially methylated regions and high methylation intensity detected in such areas, as previously mentioned. The *FABP5, PAX9* and *FKBP1B* genes were chosen among 29 candidates because they were representative of these criteria and particularly of each of the assessed genomic zones. We also selected the *CFD* gene, which singularly exhibited a methylated area in each tumor cell line (Additional file 6: Table 1).

Validation through bisulfite sequencing of *FABP5* did not lead to conclusive chromatograms that enabled us to identify differential methylation profiles, given that we identified a repetitive guanine background with similar intensity levels to both evaluated cell lines (Additional file 1: Fig. 1). The analysis of *CFD* showed two differentially methylated areas identified by WGBS, which were designated as Area 1 (differentially methylated in A2780) and Area 2 (differentially methylated in OVCAR3). Validation of Area 1 yielded no methylation in the A2780-R phenotype but did show methylation in the non-neoplastic DNA from saliva, ovary control and peripheral blood mononuclear cells (PBMCs) and in the tumor lines HeLa and LoVo (Additional file 2: Fig. 2). However, the analysis of Area 2 confirmed the *β* values ​​applied for the differential methylation identification despite only two hemimethylated positions being observed in resistance. Similar to Area 1, the DNA methylation in non-neoplastic tissues was also observed in the HeLa and LoVo tumor lines (Additional file 3: Fig. 3).

**Fig. 3:**
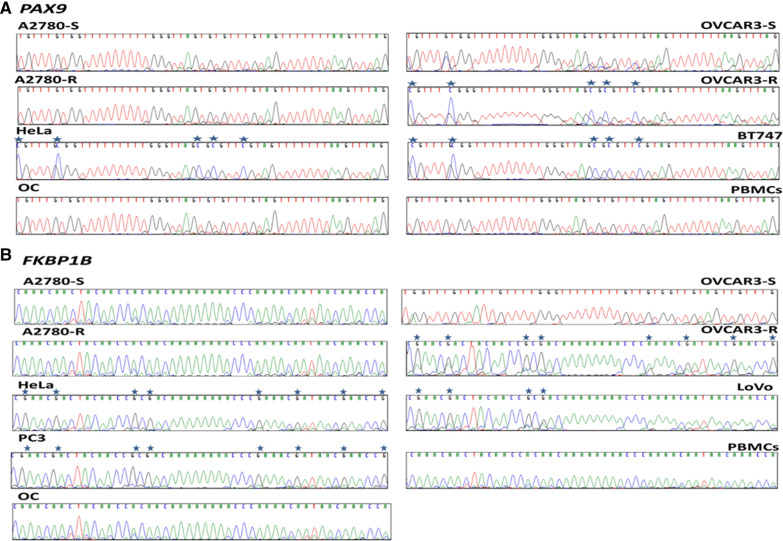
Bisulfite sequencing of *PAX9* and *FKBP1B* regulatory CGIs. Representation of ​​bisulfite-modified DNA fragment of both candidate genes from the sensitive and resistant tumor lines A2780 and OVCAR3, as well as DNA obtained from healthy ovarian tissue (OC) from patients undergoing sex change, DNA from human peripheral blood mononuclear cells (PBMCs) and from the tumor lines HeLa, BT747, LoVo and PC3. Samples were sequenced with sense primer for *PAX9* and antisense for *FKBP1B* except in OVCAR3-S. Asterisks indicate methylated positions

No methylation was detected in the ovarian and PBMC tissue control in *PAX9* or *FKBP1B* (Fig. 3), whereas clear differential methylation was identified for both in OVCAR3-R (Fig. 3), and the sensitive line exhibited no methylation. These findings confirm the WGBS results for these two candidates, given that CpG methylated positions were identified only in the OVCAR3-R cells and not in A2780-R cells, thereby validating the re-evaluation of selected *β* parameters (Additional file 6: Table 1). The *PAX9* analysis yielded methylation in HeLa and BT747 (Fig. 3A), and methylation of *FKBP1B* was also detected in HeLa, PC3 and LoVo (Fig. 3B).

### Gene expression of *PAX9* and *FKBP1B* is under epigenetic regulation in ovarian tumor cell lines

**Fig. 4:**
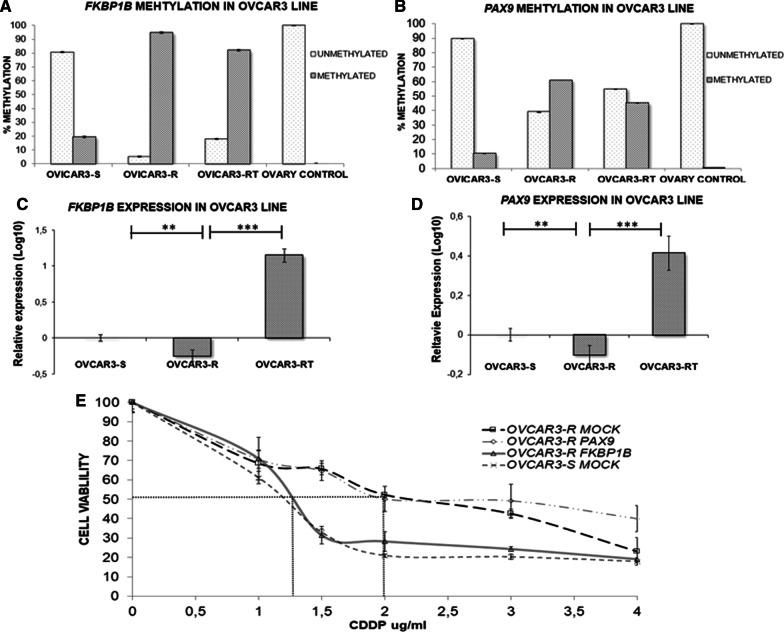
Absolute methylation and relative expression levels for *FKBP1B* and *PAX9* and the effect of overexpression of candidate genes on cell sensitivity to CDDP in the OVCAR3 cell line. **A**, **B** Representation of methylated and unmethylated molecules assessed through qMSP (quantitative methylation-specific PCR). The methylation rate obtained from three replicates for each type of evaluated sample is shown. Assays were performed in all experimental conditions: S, R and RT. S, sensitive; R, resistant; RT, resistant treated with epigenetic reactivation drugs (5-Aza and TSA). Each trial was repeated twice, and a non-neoplastic ovary sample was included as the control. **C**, **D** The expression levels of each candidate gene assessed by qRT-PCR were normalized using *GAPDH* as the endogenous control. Assays were performed in all experimental conditions of the OVCAR3 cell line. Data are represented in the log10 scale using the sensitive experimental group as a calibrator. Bars represent the mean ± SE of two independent experiments performed in duplicate. **p ≤ 0.01 ***p ≤ 0.001. **E** Viability assays of the OVCAR3 cell lines transfected with pCMV6 (S-MOCK and R-MOCK) and with the overexpression vectors (R-PAX9 and R-FKBP1B). Each experimental group was exposed for 48 h to six different CDDP concentrations, and the data were normalized to each untreated control (set to 100%). The data represent the mean ± SD of at least three independent experiments performed in quadruplicate at each drug concentration for each analyzed cell line. P-values < 0.01 indicated a significant change in drug sensitivity (Student’s t-test)

In addition to the qualitative analysis, we assessed the methylation level of *PAX9* and *FKBP1B* by implementing a quantitative approach, which included a MethylLight (quantitative methylation-specific polymerase chain reaction [qMSP]) assay. In the interrogated region of *FKBP1B*, we observed a 4.7-fold increase in methylation of contrasting resistance versus sensitivity in the corresponding cell line (95% vs. 20%) and a 13% decrease in the number of methylated molecules in the RT group compared with resistance (Fig. 4A). In the case of *PAX9*, methylation was sixfold higher (60% vs. 10%) in resistance versus sensitivity, and the RT phenotype exhibited 15% less methylation compared with the resistant phenotype (Fig. 4B). These findings suggest that cisplatin treatment induces a significant increase in the methylation rate in both genes when comparing resistance versus sensitivity. Consistent with the data from Sanger sequencing, we detected no methylated molecules in the normal ovaries for either of the two genes.

To validate the data from the gene expression microarray and determine the association between methylation and transcriptional inhibition, we performed real-time polymerase chain reaction (PCR) amplification for the two genes in the three experimental groups. *PAX9* and *FKBP1B* inhibited their expression significantly in the resistant phenotype compared with the sensitive phenotype (Fig. 4C, D). This expression pattern detected in resistance for the two candidates correlated with the methylation status inferred by WGBS, bisulfite sequencing and MethylLight. The re-expression levels of *PAX9* and *FKBP1B* identified in the RT group when compared with the resistant group (Fig. 4C, D) are indicative of the role played by epigenetic regulation, indicating that drug therapy favors the induction of gene re-expression and that transcription modulation is epigenetic-dependent for these targets in the experimental model.

### Ectopic overexpression of *FKBP1B* restores cisplatin sensitivity in resistant ovarian tumor cells

This study aimed to identify potential marker genes whose biological function grants malignant cells under treatment sufficient susceptibility for the compound to conduct its effector mechanisms. We therefore sought to demonstrate an association between the improved response to cisplatin treatment and the exogenous overexpression of our two candidate genes by conducting plasmid transfection experiments.

The viability analysis indicated that *FKBP1B* overexpression induced an increase in cisplatin sensitivity, matching up the cisplatin response values of the sensitive OVCAR3 tumor line, whereas no changes were observed in the group transfected with *PAX9*. The half maximal inhibitory concentration (IC50) was 1.3 μg/mL (SD ± 0.08) for the OVCAR3-R *FKBP1B* group and 2 μg/mL (SD ± 0.02) for the control group (OVCAR3 R-MOCK), which yielded a resistance index between these two groups of 1.53 (p ≤ 0.01) (Fig. 4E).

### *PAX9* methylation is related to decreased overall survival in cisplatin-resistant patients

**Fig. 5:**
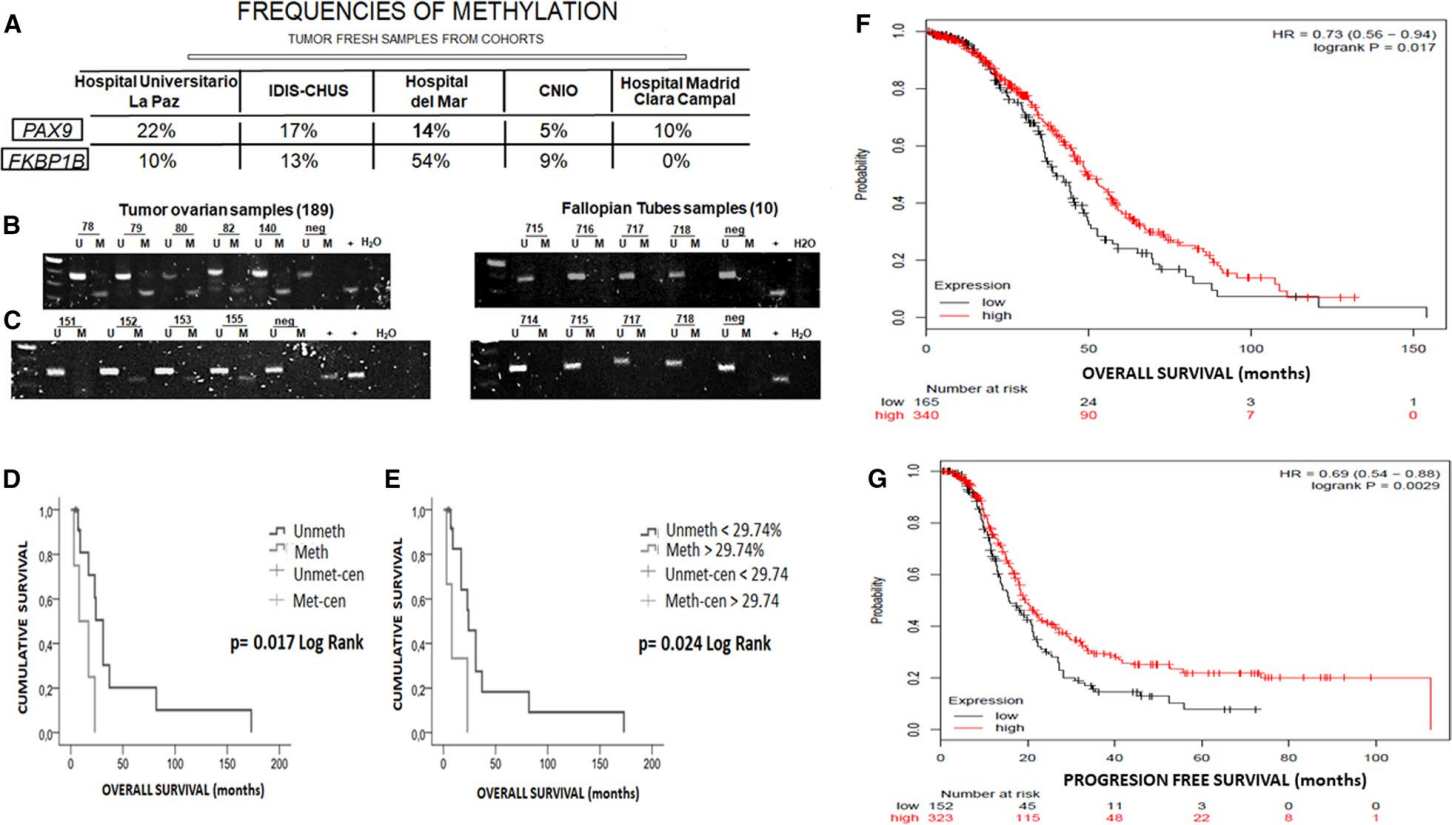
*FKBP1B* and *PAX9* methylation analysis in primary tumors and survival analysis. **A** The methylation rates were evaluated in clinical samples from different cohorts through MSP (methylation-specific PCR). For each sample, the presence of a product in lane M was considered as methylated DNA, while the product amplification in lane U was considered as non-methylated. Negative and positive controls for each reaction corresponded to DNA from PBMCs and in vitro methylated DNA, respectively. Representative MSPs of candidate genes are shown. **B** Samples 78, 79, 80, 82 and 140 showed methylation for *FKBP1B,* **C** and samples 152, 153 and 155 showed methylation for *PAX9*. Kaplan–Meier comparison in terms of overall survival (OS) between cisplatin response and *PAX9* methylation in 21 patients with platinum-resistant ovarian cancer treated. Qualitative and quantitative methylation comparisons correspond to **D** and **E**, respectively. Log-rank tests were used for comparisons, and *p* values < 0.05 indicated a significant change in OS. We employed a Kaplan–Meier plotter tool to compare *PAX9* expression versus OS and progression-free survival (PFS) (**F**, **G**) in 505 and 475 selected TCGA patients, respectively. A log-rank test was applied for comparisons, and *p* values < 0.05 indicated a significant change in OS

After studying the associated profile of these genes regarding cell viability to cisplatin, we focused on assessing their potential involvement in the therapeutic response by evaluating primary tumors. The methylation rates for *PAX9* were similar in the three cohorts, with the lowest value corresponding to the Spanish National Cancer Research Center (CNIO) group (Fig. 5A). In terms of *FKBP1B* methylation, the Hospital del Mar cohort showed the highest frequency of methylation, and not one methylated sample was found in the resistant/refractory patients (Fig. 5A). To rule out imprinting, we also tested 10 fallopian tube samples and found no methylation (Fig. 5B, C). To confirm whether methylation or the expression degree of the candidate genes influenced the clinical outcome, we performed a cross-linking analysis between gene methylation/expression and OS/PFS for each clinical parameter in the fresh tumor cohorts. There were significant differences in OS associated with *PAX9* methylation in the patients considered cisplatin-resistant. The Kaplan–Meier analysis showed that resistant/*PAX9*-methylated patients died earlier than those in whom methylation was not detected. We assessed methylation through qualitative and quantitative methods, and when relating the results from both techniques, we observed that the lowest methylation rate associated with a positive result by MSP was 29.74%. Patients with values ​​above this threshold were therefore considered methylated. There was no significant relationship with *FKBP1B* in any analysis of either expression or methylation in any cohort. To extrapolate data collected from our survival study, we applied the Kaplan Meier plotter [20], a web-based tool that uses patient information stored in the TCGA and Gene Expression Omnibus (GEO) databases. This tool performs survival analyses based solely on gene expression. We therefore explored the expression of *PAX9* in a large group of patients with ovarian cancer treated with platinum compounds. These analyses showed significant differences in the patients with low *PAX9* expression in terms of shorter survival ranges and recurrent disease compared with those that exhibited higher expression. Figure 5D, E show the influence of *PAX9* expression, with the statistical significance demonstrated through the log-rank test (*p* = 0.017 for the OS analysis and *p* = 0.0029 for PFS). Such interactions show a direct effect on gene functionality, which is reflected in the clinical landscape, showing a worsening prognosis for patients with a hypermethylated and downregulated *PAX9* gene.

Clinical information for each of the patients in each evaluated cohort, along with the results of the methodological approaches employed, are described in Additional file 13: Excel database.

### The combination of omics and clinical database resources provides potential diagnostic predictive candidates

**Fig. 6:**
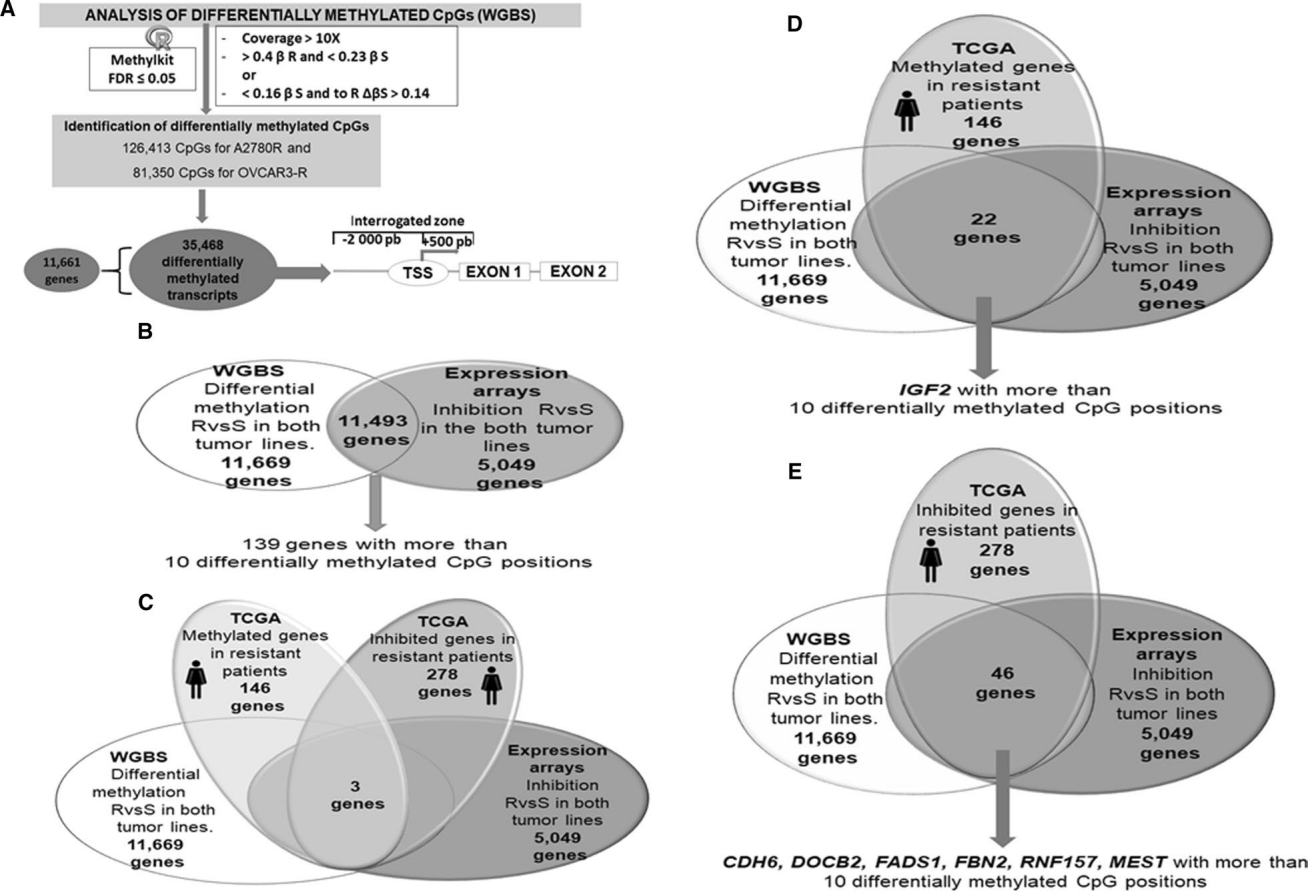
Identification of differentially methylated CpG dinucleotides through methylome sequencing and representation of cross-linked studies. **A** Data derived from whole genome bisulfite sequencing (WGBS) conducted in the ovarian cancer cell lines were analyzed by means of an FDR < 0.05, and two ranges of *β* values were implemented, one of them similar to that used in the first differential methylation analysis (*β* > 0.4 in R and < 0.23). Two additional *β* values were added to establish greater restrictiveness in sensitive samples, in which we expected to identify unmethylated cytosines (*β* < 0.16 in S) but allowing lower *β* values ​​in resistance (Δ*β* of 0.14 between R and S) to capture small tendencies of methylation in resistance. These methylated positions were interrogated in typical promoter regions. **B**, **C**, **D**, **E** Representative Venn diagrams showing the candidate genes obtained from the cross-analysis of in vitro (methylome sequencing, expression arrays) and in silico information (data related to methylation and gene expression of selected patients from the TCGA). The results of these studies show the common genes derived from the assessed parameters. The other studies can be found in Additional file 12

The lack of predictive tools based on omic technologies with the potential to assess the behavior of several genes in ovarian cancer prompted us to develop a predictive bioinformatic matrix. We established a series of computational approaches based on multiple combinations between high-throughput screening techniques such as methylation arrays, whole methylome and RNA sequencing and information from the TCGA database. In this case, we implemented two ranges of *β* values ​​to evaluate the WGBS data. These settings, along with a covering range of 10X, identified 11,661 genes with at least one differentially methylated CpG position in areas covering from 2000 base pairs upstream to 500 downstream from the transcription start site of all coding transcripts, thereby obtaining 35,468 different transcripts differentially methylated in the in vitro model (Fig. 6A).

To exploit the information that high-throughput techniques can provide, we interrogated the methylation state over 450,000 CpG positions by performing the Illumina Methylation 450 K array in fresh tumor samples, thereby obtaining 6421 differentially methylated CpG positions among the tested samples and normal controls. To determine the state of global gene expression, we performed transcriptome sequencing in the healthy tissue samples, such as ovary and fallopian tubes, and in the tumor tissue of various cohorts. One of these transcriptomically evaluated cohorts was also analyzed at the methylation level through the Illumina array.

### Multiple bioinformatic approaches contribute to enriching the predictive matrix

Several cross-studies were conducted among the methodologies mentioned above to identify relevant genes according to their methylation profiles, expression and possible suppressive role, taking into account their comparative profile between in vitro and translational observations. In this case, the TCGA patient selection criteria included treatment with platinum derivatives in all cases, availability of expression and methylation data obtained from RNA-seq and 450 K array, respectively, and a relapse within 6 months. Thirty-five patients were selected from the TCGA, 11 of whom were considered sensitive and 24 of whom were considered resistant.

Twenty-four studies were designed to identify the main potential candidates to be included in the matrix. To describe these multiple analyses rationally, we grouped them into three types according to the methodologies applied. An example of these studies is represented in Fig. 6. Group 1 comprised WGBS and expression microarray data plus information from the TCGA patients. The cross-analysis against methylation and expression yielded 11,493 hypermethylated and inhibited genes present in both resistant tumor lines (Fig. 6B), 139 of which had more than 10 methylated CpG positions (Additional file 7: Table 2), among them, the *MEST* gen. When analyzing the in vitro models along with TCGA patient information, only three common genes were found between the patients and cell lines (Fig. 6C). Among the six genes obtained from the analysis in this study, *MEST* became a relevant candidate because it was also identified in four subsequent analyses (Additional files 4, 5: Figs. 4D, 5B, 5F and 5J and Additional file 8: Table 3). Additional file 4: Fig. 4 shows a Venn diagram of group 2 related to the crossover between 450 K methylation and RNA-seq of the studied cohorts plus the study of TCGA patients, Additional file 5: Fig. 5 shows a Venn diagram for group 3, consisting of the analysis of massive methylome sequencing, expression microarrays, 450 K methylation analysis and RNA-seq of our patients. Additional file 9: Table 4 also presents a list of 73 genes with more than 10 methylated positions that originate from the analysis I shown in Additional file 5: Fig. 5.

### *MEST* as a potential biomarker in resistance to platinum treatment

**Fig. 7:**
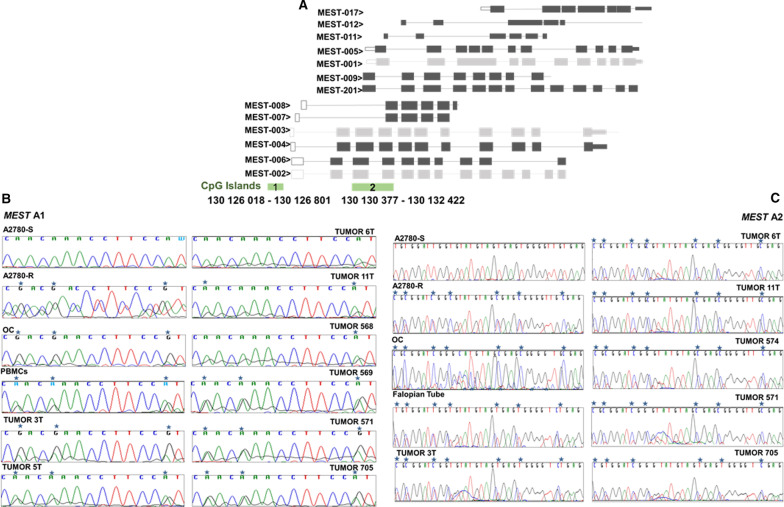
*MEST* transcript illustration and bisulfite sequencing of two potentially regulatory CpG-rich areas. **A** Island 1 is located in a typical promoter zone with the capacity to regulate the closest transcripts and possibly distant ones (Cr 7: 130 126 018—130 126 801 identified in A2780-R). A region of 529 bp in length was sequenced. Island 2, which is located in the gene body, could fit within a promoter region for transcripts 201, 9, 1, 5, 11 and 12 and possibly for transcript 17 (Cr7: 130 130 377–130 132 422, identified in A2780-R). An area of 429 bp in length with 20 CpG positions was selected to be sequenced. **B** Island 1. Representation of a sequence fragment of the ​​bisulfite-modified DNA *MEST* gene area from sensitive and resistant tumor lines A2780, normal ovarian tissue from patients undergoing sex change, DNA from peripheral blood mononuclear cells (PBMCs) and tumors from fresh tissue. All samples were sequenced with the antisense primer. **C** Island 2. Representation of a sequence fragment of the ​​bisulfite-modified DNA *MEST* gene area. In addition to the samples sequenced for the evaluation of area 1, we added a fallopian tube sample and tumor 754. PBMCs, tumors 568, 569 and 5T were not evaluated for this region. All samples were sequenced with the sense primer. Methylated positions are indicated by a blue asterisk

In the series of studies, a group of genes was characterized by exhibiting more than 10 methylated CpG positions, as well by recurrence in the resulting data. Within this particular group of genes, *MEST* was the most frequently represented gene. According to these analyses, *MEST* is methylated/inhibited in resistance in the experimental model, inhibited in platinum-resistant TCGA patients and hypomethylated and overexpressed in the tumors of patients belonging to La Paz Hospital and Santiago de Compostela cohort. This gene is comprised of 13 protein-encoding transcripts with two identified CpG-rich regions, whose methylation could be regulating the expression of its transcripts (islands 1 and 2). In our study, however, we found differential methylation exclusively in a region linked to CpG island 2 (Fig. 7A). This region could act as a possible regulatory zone for the expression of certain transcripts of *MEST*, although it is not located in the canonical promoter region. Island 2 has a greater extent than island 1 and is located at 4000 bp in the 3' direction (Fig. 7A).

### The in silico *MEST* CpG 1/2 island methylation matches the in vitro assessment

We performed bisulfite sequencing on both CpG islands in the tumor cell lines, control samples and tumor tissue from the analyzed cohorts to corroborate the *MEST* methylation status identified by our methodological combinations. Sanger sequencing yielded differential methylation in island 1 for the A2780-R line and the presence of methylation in the non-tumor samples (normal ovary, PBMCs) (Fig. 7B). Hemimethylation was also observed in all analyzed tumors, except in the 6T sample where the cytosine signal was very weak (Fig. 7B).

Differential methylation in island 2 was also validated through Sanger sequencing in the resistant tumor line, and methylated CpG positions were identified in the ovarian and fallopian tube control samples. Methylation in this area was detected in all analyzed tumors (Fig. 7B).

Based on the data from RNA-seq and 450 K array methodologies, we performed a cross-analysis to determine the influence that the degree of methylation in such areas can exercise on the regulation of MEST transcript expression. According to the *β* values and transcript count (Additional file 10: Table 5), higher methylation in island 2 than in island 1 was detected in all samples. Decreased methylation in island 1 compared with the control sample agrees with the overexpression of MEST transcript-006 in 50% of the analyzed tumor samples, while the increased methylation on shore island 1 (shore 1) correlates with the inhibition of MEST transcript-007 expression in all tumors (Additional file 10: Table 5). The analysis of island 2 showed the highest degree of methylation on island and shore areas (shore 2) in two tumor samples, those that exhibited inhibition in the expression of MEST transcript-001 (Additional file 10: Table 5).

## Discussion

Epigenetic disruptions reported in EOC related to DNA hypermethylation have been associated with the inactivation of almost all pathways involved in cancer, including DNA repair mechanisms, cell cycle regulation and apoptosis. Varying degrees of methylation have been found in numerous genes in EOC, but few are related to acquired resistance to platinum treatment. We sought to identify novel epi-biomarkers capable of predicting the behavior of the disease post-therapy by combining an experimental model with an analysis performed on patient samples and combined with public information and omic data. We established the ovarian cancer cells A2780R and OVCAR3R, with a cisplatin RI in accordance with the previously established H23R and H460R cell lines [20]. The expression analysis showed that 48% of the genes were differentially expressed in the resistant phenotypes in both tumor cell lines. Of the 9736 genes differentially expressed in this experimental model, 75% matched with altered genes observed in the data from TCGA patients.

Aberrant methylation in cells continuously exposed to cisplatin has been reported to affect the sensitivity of tumor cells to antineoplastic agents, altering the expression of crucial genes in response to this drug [21]. This high percentage of differentially expressed coincident genes suggests a similarity in treatment response between the experimental and translational scenarios. Regarding the methylome sequencing analysis, we applied a binomial analysis that resulted from the Illumina array 27 K known as the *β* value [22]. The first set of *β* values did not allow us to detect differential methylation in the initial evaluated candidates. These preliminary outcomes could have originated from the bias identified in previous studies related to the performance of massive sequencing associated with the guanine–cytosine content [23, 24]. We therefore considered this initial approach as a precedent for adjusting new parameters intended to reduce the above-mentioned bias. By using bisulfite sequencing as an alternative methodology, we found differential methylation in 50% of the evaluated targets due to rearrangement of *β* values. However, bisulfite sequencing does not have sufficient sensitivity to identify specific epigenetic signatures in formalin-fixed paraffin-embedded (FFPE) samples. In fact, this methodology cannot be used routinely in clinical practice due to its complexity and the type of samples analyzed in hospitals, given that a large percentage of cases involve paraffin samples. It is therefore necessary to explore the area of ​​epigenetic interest in additional human tumor cell lines to confirm the positions identified by next-generation sequencing that remain frequently methylated in cancer. Our bisulfite sequencing results not only confirmed the *β* values but also allowed for the design of high-sensitivity primers and double probes for these specific positions (by MSP or qMSP), ensuring their use in not only determining the percentage of demethylation after epigenetic reactivation treatment in our cellular model but also in patient paraffin samples, which supports the potential diagnostic use of the assay, as is the case for the FDA-approved epigenetic marker O6-methylguanine-DNA methyltransferase (*MGMT*) for routine clinical practice [25].

Using this double probe approach (qMSP), the demethylation rate observed after the epigenetic reactivation treatment in our “in vitro” model agreed with that of previous studies and ranged from 14 to 30% [26, 27]. The demethylation rate observed for *PAX9* and *FKBP1b* genes significantly upregulated their expression levels, as reported for other candidates, with a 70-fold to more than 270-fold increase after the same epigenetic reactivation treatment [18, 27, 28]. It is also possible that this strong increase in expression levels could be due to histone acetylation. According to an article by Kuznik et al*.*, there are peptides that can bind to the histone H1.3 protein, altering its conformation, causing changes in the chromatin structure in the loci of certain genes, in particular *FKBP1b* [29].

These changes could alter gene expression in extreme biological situations such as chemotherapy and the use of epigenetic pharmacological reactivation drugs. Nevertheless, we were able to validate the methylation of our markers both qualitatively and quantitatively, as well as the absence of methylation in control samples. Therefore, optimization of *β* values ​​contributed to the identification of genes with differential methylation in the cellular model of platinum resistance.

A direct interaction has been reported between hypermethylation in promoter regions and genomic silencing in cancer [30, 31], but the role of regions located distally to the canonical CpG islands in regulating gene expression has only recently been determined. Less common is the interrogation of methylation in intragenic zones and its association with controlling gene expression [32]. Differential methylation on *PAX9* was identified in a region located 4 kb bp upstream of the promoter region. We observed an inverse relationship between high methylation levels and low *PAX9* expression in the OVCAR3-R group, a finding that highlights the influence of hypermethylation in distal areas. *FKBP1B* exhibited a methylated area within the gene and just like *PAX9.* An inverse relationship between methylation and expression was identified in vitro. It has been suggested that DNA methylation in the gene body can increase the transcriptional activity by blocking the initiation of intragenic promoters [33]. However, other studies have reported an inverse association between expression and methylation regions such as exons and introns [34, 35]. Zhang et al. demonstrated that *PMP24*, a tumor suppressor gene, is silenced in prostate cancer lines through methylation of a CpG island that overlaps part of the promoter, exon 1 and part of the first intron [36]. This finding is similar to the epigenetic and transcriptional trend we identified in *FKBP1B *in vitro, even in relation to the assessed CpG island, because although our region is located specifically in intron 1, it belongs to a larger island that encloses exon 1 and the promoter of this gene, just like *PMP24.* In terms of biological roles, the ectopic expression of *FKBP1B* in the resistant phenotype promoted a decline in cell survival after CDDP treatment, mimicking the behavior of OVCAR3-S. However, *PAX9* overexpression had no effect on cell survival. We have left the door open to developing future assays with *PAX9* under different transfer conditions, mainly due to the features observed in this gene in the translational model, such as an association regarding methylation and survival in CDDP-resistant patients in terms of longer survival in those who without the methylation. OS is considered the variable of greatest confidence and interest when the effects of an intervention are to be analyzed [37]. Despite not finding a similar relation in terms of *PAX9* inhibition, we performed a survival analysis through a web tool using data from the *TCGA* database. This approach showed that low *PAX9* expression has a negative effect on OS and PFS, thereby confirming that *PAX9* exhibits a desirable profile as an epi-biomarker. *PAX* genes (paired box) are transcription factors that contain a highly conserved DNA binding domain called paired domain [38], which regulates processes such as proliferation, resistance to apoptosis, cell migration and invasion [38]. To date, only one study has described the role of *PAX9* as a predictive marker in cancer. Tan et al. (2017) observed an interaction between the expression of this gene and postoperative radiotherapy in esophageal squamous cell carcinoma, wherein patients with high expression showed greater life expectancy and delayed disease reoccurrence, granting this gene a positive role as a marker of radiosensitivity in this type of malignancy [39].

The outcomes of the survival analyses performed for *FKBP1B* resembled those for *PAX9* but without statistical significance, probably due to the low number of methylated patients (4 of 49). However, Tougeron et al*.* proposed a possible association between the low frequency of mutation in known oncogenes such as *KRAS* (< 10%) and the response to colon cancer treatment [40]. It is likely that a similar situation occurs for *FKBP1B* and that increasing the sample size would lead to the identification of a translational role for this gene, as with *PAX9*. FK506-binding proteins (FKBPs) are intracellular ligands of FK506 and rapamycin. This family of proteins is involved in the intracellular release of calcium, gene transcription, protein translation and cell trafficking [41]. Complexes formed between FKBPs and their ligands regulate cell signaling pathways, including the mechanistic target of rapamycin (mTOR) [42], and hyperactivation of this molecule is known to play a role in tumor transformation and growth. It was believed that only *FKBP12* acts as a link between rapamycin/FK506 and mTOR; however, *FKBP1B* has been identified as another mediator in this interaction and therefore has the potential to inhibit mTOR kinase activity [43]. Liu et al. demonstrated that the FKBP12 protein promotes the degradation of the oncogenic protein MDM2 through self-ubiquitination, favoring the efficiency of doxorubicin treatment in tumor lines of neuroblastoma [44]. It has also been reported that inhibition of the member* FKBP51* leads to increased resistance in chemotherapy of several tumor cell lines [45]. So far, there is no solid evidence of the role that *FKBP1B* could play in resistance for any malignancy. Our study therefore provides a first insight into the function and influx that our candidates can exert on the response to conventional treatment in ovarian cancer.

Between 1971 and 2007, survival in ovarian cancer increased by only 17%, while the 10-year survival for breast cancer increased 38% [37]. Only a few markers have been identified and put into clinical practice in EOC. We therefore focused on identifying potential differentially methylated transcripts by epigenetic interrogation in canonical promoter regions to identify key markers, supported by interpolation of several approaches. We submitted this data to all possible comparisons to simulate the potential information that could be derived from the in silico performance of a constantly enriched matrix. Among the potential targets originating from these tests, we chose *MEST* to corroborate our study design because of its previously described predictive epi-role in EOC [46]. Sanger sequencing helped verify the accuracy of the *β* values ​​selected for this second goal, given that methylation was not observed in the sensitive phenotype of line A2780 but was observed in the resistant parental line. Differential methylation in the validated line A2780 was also identified by Zeller et al. but over a different region (island 1). The authors also demonstrated *MEST* re-expression by epigenetic treatment demonstrating the regulatory mechanism. This evidence reinforced the direction and approaches we employed in our search for candidates. *MEST* is regulated by genetic imprinting and deregulation of methylation, and its expression has been associated with invasive breast cancer [47], invasive cervix cancer [48] and the onset of lung adenocarcinoma [49]. Imprinting can exert a promoter-specific regulation, as reported by Pendersen et al., who identified that transcript 1 of *MEST* is underimprinting in control samples and tumor tissue in invasive breast cancer, thereby maintaining its monoallelic expression, while transcript 2 is biallelically expressed in most tumors [50]. We compared the methylation in both islands and shore regions (from the study by Zeller et al. and from our study) and found that the monoallelic expression of this MEST-001 transcript is possibly related to the maintenance of imprinting in area 2. Our results indicate that MEST-001 inhibition could be related to the chemotherapy response and that this transcript regulation governs the methylation balance of the region examined in our study. The effects of these mechanisms in *MEST* have been related to the biology of certain tumors, but until now, no study has evaluated this behavior in such a specific approach regarding treatment resistance.

Although the observed characteristics of *MEST* make it a candidate predictor gene, *PAX9* is the main output from the model proposed in this study, having demonstrated its epigenetic characteristics in vitro in addition to the direct relationship between its methylation and the poor prognosis of the platinum-resistant study patients.

## Materials and methods

### Cell lines, platinum viability and epigenetic reactivation treatment

Six human cancer cell lines were purchased from ATCC (Manassas, VA) and ECACC (Sigma-Aldrich, Spain) and cultured according to the recommendations. The CDDP-resistant variants A2780R and OVCAR3R were established in our group according to a protocol developed for H23R and H460R variants [51]. The ovarian-resistant types were selected after a final exposure to 0.5 (A2780R) and 0.05 (OVCAR3R) μg/mL cisplatin (Farma Ferrer, Spain). An additional 4 tumor cell lines (PC3, BT474, LoVo and HeLa) were used to validate the most frequent methylated positions identified by bisulfite sequencing. The platinum compound’s effect on cell viability was assessed by exposing the tumor cell lines to increasing doses of CDDP, as previously described [52]. To achieve gene re-expression, the resistant variants were epigenetically treated by exposing them to 5-aza-2'-deoxycytidine (5Aza-dC) and trichostatin A (TSA) (Sigma-Aldrich, Spain) at concentrations of 5 μM and 300 nM, respectively, as previously described [53].

### Clinical sample and data collection

We collected fresh frozen and FFPE ovarian cancer samples along with the clinical data from various institutions in Spain. Frozen samples representing the most frequent ovarian cancer subtypes were obtained from La Paz University Hospital (HULP) (10 patients) and from the Health Research Institute-University Hospital Complex of Santiago de Compostela-HULP Biobank (47 patients). FFPE samples were provided by Hospital Parc de Salut Mar (83 patients), 7 samples from patients categorized as stage III/IV with a platinum treatment response were provided by Hospital Madrid Clara Campal, and 39 high-grade serous carcinoma (HGSOG) samples were obtained from CNIO Biobank. We also collected DNA from various tissues to rule out gene imprinting. DNA was therefore extracted from PBMCs from 4 healthy donors, as well as from saliva and healthy ovarian samples from individuals who had undergone sex reassignment surgery. We also extracted RNA from 10 fallopian tube samples from women undergoing tubal ligation. Follow-up was conducted according to the criteria of the medical oncology divisions of each institution. The clinical parameters associated with the study included age, carcinoma type, histology grade, stage, treatment, OS and overall progression-free survival.

### Gene expression assays

Gene expression microarrays were conducted on various experimental tumor cell line groups, as previously described [53]. Total RNA for quantitative RT-PCR was isolated from non-neoplastic tissue and tumor cells using Trizol (Life Technologies, Rockville, MD, USA) according to the manufacturer’s instructions. RNA was retrotranscribed, and a quantitative analysis was performed, as previously described [51, 53]. Samples were analyzed in triplicate using the HT7900 real-time PCR system (Applied Biosystems, USA), and relative expression levels were calculated according to the comparative threshold cycle method (2^−ΔΔCt^) using *GAPDH* as an endogenous control gene. Probes for gene expression (Applied Biosystems, USA) were as follows: *PAX9:* Hs00196354_m1; *FKBP1B*: Hs00997682_m1; and *GAPDH:* Hs03929097_g1. Microarray and qRT-PCR assays are detailed in Additional file 12: Materials and Methods section. Each study included control samples as calibrators to reference the results.

A transcriptome study was performed through RNA-seq technology based on RNA extraction from frozen fresh samples encompassing non-neoplastic cells and tumor cells. Total RNA was isolated from controls and tumor samples and delivered to *Fundacion Centro Nacional de Investigaciones Cardiovasculares* (National Center Foundation for Cardiovascular Research, CNIC) and *Sistemas Genomicos* (Genomic Systems, ASCIRES), to be processed by their respective protocols and sequenced with Ilumina Hseq 2500 technology. The detailed process is described in Additional file 12.

RNA-seq provided a view of the gene expression through transcript quantification, given that the output reads were individually mapped to the source genome and counted, thereby obtaining the density corresponding to each known exon of our gene candidates.

### Epigenetic validation

DNA from human cancer cell lines, ovarian specimens and non-neoplastic tissues were isolated, bisulfite-modified as described [53] and then employed for bisulfite sequencing, MSP, qMSP, WGBS assays and 450 K array. The WGBS and DNA extraction protocols for each type of sample are described in Additional file 12: Materials and Methods section, as well the PCR settings and primer sequences for the BS, MSP and qMSP probes (Additional file 6 and 11: Tables 1 and 6). The features of the genomic regions selected for epigenetic validation can also be found in Additional file 6: Table 1. qMSP is based on qPCR methodology, which allows for quantitative differential methylation assessment due to the simultaneous presence of both methylated and non-methylated detection probes, each of which is tagged with a different dye, and is therefore a multiplex strategy. We developed the assay through the QuantitTect Multiplex PCR kit (Quiagen, the Netherlands), and in-house designed probes were synthetized by Applied Biosystems (USA). Fresh frozen samples were analyzed in duplicate using the HT7900 real-time PCR system (Applied Biosystems, USA). We also performed a high-throughput methylation analysis through the HumanMethylation450 BeadChip array. Briefly, we obtained DNA from 5 frozen samples, 1 healthy ovarian tissue sample and 4 tumor samples belonging to the HULP cohort, which were also transcriptome sequenced. The DNA was extracted according to the aforementioned procedures and sent to CNIO for the array procedure. The HumanMethylation450 BeadChip array has a capacity of 12 samples and interrogates more than 450,000 CpG sites using Infinium HD Methylation technology.

### cDNA plasmid transfection

The experimental tumor cell line groups were transfected with test cDNA plasmids to determine their potential biological role. A Myc-DDK-tagged ORF clone of *PAX9* (ID: RC200380), *FKBP1B* (ID: RC200667) and the negative control pCMV6 were used for transfection (OriGene, USA). OVCAR3 S/R phenotypes were seeded onto 60-mm dishes at 5 × 10^5^ cells/dish density and transfected with either a negative control or test vector using jetPei (Polyplus-transfection, Graffenstaden, France) as a transfection agent according to the manufacturer’s instructions. Seventy-two hours after transfection, the cells were exposed to increasing doses of the platinum compound, and the survival fraction was calculated following the previously described method [52]. Transfection efficacy was evaluated by measuring cDNA plasmid gene expression at 24 and 72 h by qRT-PCR as described above, using the resistant cell line transfected with negative control as a calibrator. Three independent experiments were performed in triplicate.

### Computational analysis

#### Epigenetic study

Regions found to be differentially methylated by the WGBS assay were identified as CpG islands using informatics tools. To this end, we applied search engines that use strategies based on Takai and Jones parameters: GC ≥ 55%; Obs/Exp ≥ 65; and length ≥ 500 bp [54]. We applied two search engines linked to websites (http://bioinfo.itb.cnr.it/cgi-bin/wwwcpg.pl and http://doua.prabi.fr/software/cpgprod_query) that identify promoters associated with CpG islands in large genomic regions, exhibiting high sensitivity and specificity [55]. To increase the specificity of the search, the sequences were processed with the RepeatMasker Web Server (http://www.repeatmasker.org), discarding experimental background that might originate by repetitive ALU elements with structures similar to the CpG islands [54, 56]. These positions were confirmed by data from the ENCODE project (http://www.genome.ucsc.edu/index.html).

The data obtained from 450 methylation arrays were evaluated through the minfi [57] and ChAMP [58] packages, which help obtain quality controls, filtering, normalizations and identification of differentially methylated CpG sites. Libraries were paired-end sequenced following guidelines on the HiSeq2000 (Illumina, Inc.), with a reading length of 2 × 101 bp. Image analysis, assignment of bases and score quality of the run were processed with the real-time analysis program according to the manufacturer’s instructions.

Output data related to the differential methylation from the WGBS and arrays were obtained through statistical packages in synergy with *β* values, applied in Illumina microarrays to discriminate methylation levels [59]. Potential differentially methylated regions were evaluated at first glance with the *β* value parameter, and those that fit in the established ranges were analyzed with the methylKit package [60] to identify those with statistical significance.

#### Transcriptome study

Regarding the bioinformatic expression analysis, the Limma statistical package [61] helped identify the differentially expressed genes from the microarray analysis using the unpaired t-test algorithm with Benjamini Hochberg as the FDR correction method for multiple testing corrections. RNA-seq reads were processed through a workflow based on FastQC to ensure quality and through Cutadapt v1.3 [62] to eliminate remnants of the Illumina adapters and to discard sequences shorter than 30 bp. Reads were analyzed through the quantification of genes and isoforms methodology RSEM v1.2.3 (RNA-Seq by Expectation Maximization) [63]. A differential expression analysis was conducted using the edgeR package [64] to obtain genes/transcripts differentially expressed in tumor tissue versus normal tissue (FDR < 0.05). Those genes/transcripts with fewer than 1 read per million in fewer than 6 samples were discarded. Normalization was performed using the trimmed mean of M-values method [65], and the applied model was QLF, taking into account the technical variability related to the fact that the samples were sequenced in different centers and phases.

### Statistical analysis

The clinical data were statistically evaluated using a Chi-squared test or Fisher’s exact test for qualitative variables and Student’s t-test or the Wilcoxon–Mann–Whitney test (non-normal distribution) for quantitative variables. The Kaplan–Meier method was employed to plot the cumulative OS curves for the platinum-resistant patients with methylated or non-methylated genes according to the log-rank test. Statistical significance was defined as a bimodal p-value < 0.05. The statistical analyses were performed using Stata 10 and R 3.6 software.

## Supplementary Information

**Additional file 1**. **Supplementary Figure 1.** Bisulfite sequencing of FABP5 gene. Representation of a sequence fragment from the FAPB5 gene area of bisulfite-modified DNA from sensitive and resistant A2780 and OVCAR3 tumor lines, DNA from normal ovarian tissue from patients undergoing sex change, and DNA from Peripheral Blood Mononuclear Cells (PBMCs) and tumor lines A431 and HeLa. All samples, except Hela, were sequenced with the antisense primer. Methylated positions are indicated with a blue Asterisk.

**Additional file 2**. **Supplementary Figure 2.** Bisulfite sequencing of the CFD gene Area1. Representation of a sequence fragment of CFD gene of bisulfite-modified DNA from sensitive and resistant tumor lines A2780, DNA normal ovarian tissue from patients undergoing sex change and from Peripheral Blood Mononuclear Cells (PBMC's) as well DNA extracted from oral epithelium. The sequenced tumor lines were cervical cancer (HeLa) and adenocarcinoma of the colon (LoVo). All the samples were sequenced with the sense primer. Methylated positions are indicated by a blue asterisk.

**Additional file 3**. **Supplementary Figure 3.** Bisulfite sequencing of the CFD gene Area 2. Representation of a sequence fragment of the CFD gene of bisulfite-modified DNA from the sensitive and resistant A2780/ OVCAR-3 tumor lines, normal ovarian tissue from patients undergoing sex change, DNA from Peripheral Blood Mononuclear Cells (PBMCs) and DNA extracted from oral epithelium The sequenced tumor lines were cervical cancer (HeLa) and adenocarcinoma of the colon (LoVo). All the samples were sequenced with the reverse primer. Methylated positions are indicated by a blue asterisk.

**Additional file 4**. **Supplementary Figure 4.** Set of cross-analysis called group 2 designed to identify genes of interest. Venn’s diagrams A, B, C, D, E, F, G and H show genes derived from the analyzes developed between the Illumina 450K methylation array and transcriptome data obtained through RNA-seq performed on patient samples with the methylation and expression data of TCGA patients. It should be mentioned that in order not to omit possible candidates, the search for markers also included overexpressed and hypomethylated genes in the tumors of patients, since genes that resemble the profile sought in in vitro resistance could be found within such a group. Candidates exhibiting lower expression in R regarding another group of genes in S may correlate with hypomethylation in S (lower β values) or hypermethylation in R.

**Additional file 5**. **Supplementary Figure 5.** Set of cross-analysis called group 3 designed to identify genes of interest. Venn’s diagrams A, B, C, D, E, F, G, H, I, J, K and L show genes derived from the analyzes developed between data obtained from Illumina methylation array 450K, transcriptome data obtained through RNA-seq both performed on patient samples, with methylation and expression data from the experimental model, that is, expression array and WGBS performed in vitro. It should be mentioned that in order not to omit possible candidates, the search for markers also included overexpressed and hypomethylated genes in the tumors of patients, since genes that resemble the profile sought in in vitro resistance could be found within such a group. Candidates exhibiting lower expression in R regarding another group of genes in S may correlate with hypomethylation in S (lower β values) or hypermethylation in R.

**Additional file 6**. **Supplementary Table 1.** Bisulfite PCR amplification features of initial candidate genes. Genes with differentially methylated regions obtained by WGBS in the OVCAR3 (S/R) and A2780 (S/R) lines were validated by bisulfite sequencing and further analyzed in additional tumor lines, in order to know the methylation frequency of those CpG positions. This analysis allowed the subsequent design of specific oligonucleotides for methylated and unmethylated positions in methylation-specific PCR. Here it is also shown the chromosomal location of the region observed as differentially methylated by methylome sequencing, the cell line in which that region was identified and the number of CpG (CG dinucleotides) in which the methylation mark was found when resistance vs. sensitivity was contrasted. The amplification conditions for PCR were 5' at 95º, 40 cycles (1' at 95ºC, 1' at 60 or 62º [Annealing temperature for each gene was obtained by performing a gradient PCR], 1' at 72º and a final extension of 8' at 72ºC. PAX9 region was splitted into two areas due to its length. F: forward sense, R: reverse sense.

**Additional file 7**. **Supplementary Table 2.** Genes derived from analysis of contrast B Group 1 (Figure 6) with more than 10 positions differentially CpG methylated. MEST gen is highlighted.

**Additional file 8**. **Supplementary Table 3.** Genes resulting from contrast J Group 3 (Figure S5) with more than 10 positions differentially CpG methylated. MEST gen is highlighted

**Additional file 9**. **Supplementary Table 4.** Genes resulting from contrast I Group 3 (Figure S5) with more than 10 positions differentially CpG methylated.

**Additional file 10**. **Supplementary Table 5.** Methylation and expression cross-analysis regarding the CpG islands and shores of MEST gen. Based on the data obtained from RNA-seq and the Illumina 450K array performed in our patients, we did a cross-analysis between methylation and expression of each of these islands and their associated shore regions, with the aim of identifying the influence that the degree of methylation of these areas may exert on the regulation of the expression of MEST transcripts patients. The level of methylation in the samples was assessed by the study of the β value using the same range as that used in the screening of potential genes in the first approach. Blue color represents hypermethylation and the red color hypomethylation. The inhibited transcripts are represented in smaller size and those over-expressed in larger.

**Additional file 11**. **Supplementary Table 6.** Specific methylation amplification features of PAX9 and FKBP1B genes. Once the methylation frequencies of the different CpG positions in the tumor lines were analyzed through bisulfite sequencing, those with the highest were chosen to perform the Methylation Specific PCR technique in the different cohorts of ovarian cancer patients. PCR reactions were performed on primary tumors and control samples and amplification conditions depended on the gradient reactions performed for each of the genes, varying in both cycles and annealing temperatures. The amplification conditions for PCR were 5' at 95º, 8' at 50ºC, the number of cycles depended on each gene (1' to 95ºC, annealing temperature for each gene was obtained by performing a temperature gradient PCR. Annealing was a 1’ long and extension for 1' at 72ºC) and a final extension of 8' at 72ºC. Primers and probes used to amplify the methylated and unmethylated areas of each gene of interest are also shown. Probes are labeled with fluorophores for the quantitative determination of methylation in these genes through quantitative MSP (qMSP). F: forward sense primer, R: reverse sense primer.

**Additional file 12**. Word document containing supplementary information related to the protocols of the techniques used in the development of this study.

**Additional file 13**. Excel document containing the clinical information of the patients grouped in the different cohorts that were included for the translational phase of the study.

## Data Availability

All data generated or analyzed during this study are included in this published article (and its Additional files). The datasets used and/or analyzed during the current study are available from the corresponding author on reasonable request.

## Abbreviations

PBMCs: Peripheral blood mononuclear cells
BS: Bisulfite sequencing
CDDP: Cisplatin
CGI: CpG island
mSP: Methylation-specific PCR
OEC: Ovarian epithelial carcinoma
OS: Overall survival
PFS: Progression-free survival
qRT-PCR: Quantitative real-time polymerase chain reaction
qmSP: Quantitative methylation-specific polymerase chain reaction
RI: Resistance index

## References

[1] Jemal A, Bray F, Center MM, Ferlay J, Ward E, Forman D (2011). Global cancer statistics. CA Cancer J Clin.

[2] Wright AA, Bohlke K, Armstrong DK, Bookman MA, Cliby WA, Coleman RL (2016). Neoadjuvant chemotherapy for newly diagnosed, advanced ovarian cancer: Society of Gynecologic Oncology and American Society of Clinical Oncology clinical practice guideline. J Clin Oncol.

[3] Goff BA, Mandel L, Muntz HG, Melancon CH (2000). Ovarian carcinoma diagnosis. Cancer.

[4] Slodkowska EA, Ross JS (2009). MammaPrint^TM^ 70-gene signature: another milestone in personalized medical care for breast cancer patients. Expert Rev Mol Diagn.

[5] Clark-Langone KM, Sangli C, Krishnakumar J, Watson D (2010). Translating tumor biology into personalized treatment planning: analytical performance characteristics of the Onco type DX® Colon Cancer Assay. BMC Cancer.

[6] Cronin M, Sangli C, Liu M-L, Pho M, Dutta D, Nguyen A (2007). Analytical validation of the Oncotype DX genomic diagnostic test for recurrence prognosis and therapeutic response prediction in node-negative, estrogen receptor–positive breast cancer. Clin Chem.

[7] Kartha GK, Nyame Y, Klein EA. Evaluation of the oncotype DX genomic prostate score for risk stratification in prostate cancer patients considered candidates for active surveilance. J Clin Oncol Am Soc Clin Oncol. 2014. p. 266.

[8] Grendys EC, Fiorica JV, Orr JW, Holloway R, Wang D, Tian C (2014). Overview of a chemoresponse assay in ovarian cancer. Clin Transl Oncol.

[9] Krivak TC, Lele S, Richard S, Secord AA, Leath CA, Brower SL (2014). A chemoresponse assay for prediction of platinum resistance in primary ovarian cancer. Am J Obstet Gynecol.

[10] Muraji M, Sudo T, Iwasaki S, Ueno S, Wakahashi S, Yamaguchi S (2013). Histopathology predicts clinical outcome in advanced epithelial ovarian cancer patients treated with neoadjuvant chemotherapy and debulking surgery. Gynecol Oncol.

[11] Bast RC, Hennessy B, Mills GB (2009). The biology of ovarian cancer: new opportunities for translation. Nat Rev Cancer.

[12] Landrum LM, Java J, Mathews CA, Lanneau GS, Copeland LJ, Armstrong DK (2013). Prognostic factors for stage III epithelial ovarian cancer treated with intraperitoneal chemotherapy: a Gynecologic Oncology Group study. Gynecol Oncol.

[13] Levanon K, Crum C, Drapkin R (2008). New insights into the pathogenesis of serous ovarian cancer and its clinical impact. J Clin Oncol.

[14] Agarwal R, Kaye SB (2003). Ovarian cancer: strategies for overcoming resistance to chemotherapy. Nat Rev Cancer.

[15] Watanabe Y, Ueda H, Etoh T, Koike E, Fujinami N, Mitsuhashi A (2007). A change in promoter methylation of hMLH1 is a cause of acquired resistance to platinum-based chemotherapy in epithelial ovarian cancer. Anticancer Res.

[16] Gifford G, Paul J, Vasey PA, Kaye SB, Brown R (2004). The acquisition of hMLH1 methylation in plasma DNA after chemotherapy predicts poor survival for ovarian cancer patients. Clin Cancer Res.

[17] Matei D, Fang F, Shen C, Schilder J, Arnold A, Zeng Y (2012). Epigenetic resensitization to platinum in ovarian cancer. Cancer Res.

[18] Vera O, Jimenez J, Pernia O, Rodriguez-Antolin C, Rodriguez C, Cabo FS (2017). DNA methylation of miR-7 is a mechanism involved in platinum response through MAFG overexpression in cancer cells. Theranostics.

[19] Molaro A, Hodges E, Fang F, Song Q, McCombie WR, Hannon GJ (2011). Sperm methylation profiles reveal features of epigenetic inheritance and evolution in primates. Cell.

[20] Szász AM, Lánczky A, Nagy Á, Förster S, Hark K, Szabó A, et al. Cross-validation of survival associated biomarkers in gastric cancer using transcriptomic data of 1,065 patients. Oncotarget. 2016:49322–33. 10.18632/oncotarget.10337 PMC5226511 27384994

[21] Chang X, Monitto CL, Demokan S, Kim MS, Chang SS, Zhong X (2010). Identification of hypermethylated genes associated with cisplatin resistance in human cancers. Cancer Res.

[22] Bibikova M, Barnes B, Tsan C, Ho V, Klotzle B, Le JM (2011). High density DNA methylation array with single CpG site resolution. Genomics.

[23] Benjamini Y, Speed TP (2012). Summarizing and correcting the GC content bias in high-throughput sequencing. Nucleic Acids Res.

[24] Ji L, Sasaki T, Sun X, Ma P, Lewis ZA, Schmitz RJ (2014). Methylated DNA is over-represented in whole-genome bisulfite sequencing data. Front Genet.

[25] Rosas-Alonso R, Colmenarejo-Fernandez J, Pernia O, Rodriguez-Antolín C, Esteban I, Ghanem I (2021). Clinical validation of a novel quantitative assay for the detection of MGMT methylation in glioblastoma patients. Clin Epigenet.

[26] Yang AS, Doshi KD, Choi S-W, Mason JB, Mannari RK, Gharybian V (2006). DNA methylation changes after 5-aza-2′-deoxycytidine therapy in patients with leukemia. Can Res.

[27] Zhou Y, Hu Z (2016). Epigenetic DNA demethylation causes inner ear stem cell differentiation into hair cell-like cells. Front Cell Neurosci.

[28] Vera O, Rodriguez-Antolin C, de Castro J, Karreth FA, Sellers TA, de Caceres II (2018). An epigenomic approach to identifying differential overlapping and cis-acting lncRNAs in cisplatin-resistant cancer cells. Epigenetics.

[29] Kuznik B, Davydov S, Popravka E, Lin’kova N, Kozina L, Khavinson VK (2019). Epigenetic mechanisms of peptide-driven regulation and neuroprotective protein FKBP1b. Mol Biol.

[30] Herman JG, Baylin SB (2003). Gene silencing in cancer in association with promoter hypermethylation. N Engl J Med.

[31] Jones PA, Baylin SB (2002). The fundamental role of epigenetic events in cancer. Nat Rev Genet.

[32] Jones PA (2012). Functions of DNA methylation: islands, start sites, gene bodies and beyond. Nat Rev Genet.

[33] Maunakea AK, Nagarajan RP, Bilenky M, Ballinger TJ, D’Souza C, Fouse SD (2010). Conserved role of intragenic DNA methylation in regulating alternative promoters. Nature.

[34] Li S, Hansman R, Newbold R, Davis B, McLachlan JA, Barrett JC (2003). Neonatal diethylstilbestrol exposure induces persistent elevation of c-fos expression and hypomethylation in its exon-4 in mouse uterus. Mol Carcinog.

[35] Ortmann CA, Eisele L, Nückel H, Klein-Hitpass L, Führer A, Dührsen U (2008). Aberrant hypomethylation of the cancer–testis antigen PRAME correlates with PRAME expression in acute myeloid leukemia. Ann Hematol.

[36] Zhang X, Wu M, Xiao H, Lee MT, Levin L, Leung YK (2010). Methylation of a single intronic CpG mediates expression silencing of the PMP24 gene in prostate cancer. Prostate.

[37] Lloyd KL, Cree IA, Savage RS (2015). Prediction of resistance to chemotherapy in ovarian cancer: a systematic review. BMC Cancer.

[38] Blake JA, Ziman MR (2014). Pax genes: regulators of lineage specification and progenitor cell maintenance. Development.

[39] Tan B, Wang J, Song Q, Wang N, Jia Y, Wang C (2017). Prognostic value of PAX9 in patients with esophageal squamous cell carcinoma and its prediction value to radiation sensitivity. Mol Med Rep.

[40] Tougeron D, Lecomte T, Pagès J-C, Villalva C, Collin C, Ferru A (2013). Effect of low-frequency KRAS mutations on the response to anti-EGFR therapy in metastatic colorectal cancer. Ann Oncol.

[41] Harrar YI, Bellini C, Faure J-D (2001). FKBPs: at the crossroads of folding and transduction. Trends Plant Sci.

[42] Jacinto E, Hall MN (2003). Tor signalling in bugs, brain and brawn. Nat Rev Mol Cell Biol.

[43] Lam E, Martin MM, Timerman AP, Sabers C, Fleischer S, Lukas T (1995). A novel FK506 binding protein can mediate the immunosuppressive effects of FK506 and is associated with the cardiac ryanodine receptor. J Biol Chem.

[44] Liu T, Xiong J, Yi S, Zhang H, Zhou S, Gu L, et al. FKBP12 enhances sensitivity to chemotherapy-induced cancer cell apoptosis by inhibiting MDM2. Oncogene. 2016. 10.1038/onc.2016.331 PMC5378873 27617579

[45] Pei H, Li L, Fridley BL, Jenkins GD, Kalari KR, Lingle W (2009). FKBP51 affects cancer cell response to chemotherapy by negatively regulating Akt. Cancer Cell.

[46] Zeller C, Dai W, Steele NL, Siddiq A, Walley AJ, Wilhelm-Benartzi CS (2012). Candidate DNA methylation drivers of acquired cisplatin resistance in ovarian cancer identified by methylome and expression profiling. Oncogene.

[47] Pedersen IS, Dervan PA, Broderick D, Harrison M, Miller N, Delany E (1999). Frequent loss of imprinting of PEG1/MEST in invasive breast cancer. Cancer Res.

[48] Vidal AC, Henry N, Murphy S, Oneko O, Nye M, Bartlett J (2014). PEG1/MEST and IGF2 DNA methylation in CIN and in cervical cancer. Clin Transl Oncol.

[49] Kohda M, Hoshiya H, Katoh M, Tanaka I, Masuda R, Takemura T (2001). Frequent loss of imprinting of IGF2 and MEST in lung adenocarcinoma. Mol Carcinog.

[50] Pedersen IS, Dervan P, McGoldrick A, Harrison M, Ponchel F, Speirs V (2002). Promoter switch: a novel mechanism causing biallelic PEG1/MEST expression in invasive breast cancer. Hum Mol Genet.

[51] Ibanez de Caceres I, Cortes-Sempere M, Moratilla C, Machado-Pinilla R, Rodriguez-Fanjul V, Manguan-Garcia C (2010). IGFBP-3 hypermethylation-derived deficiency mediates cisplatin resistance in non-small-cell lung cancer. Oncogene.

[52] Chattopadhyay S, Machado-Pinilla R, Manguan-Garcia C, Belda-Iniesta C, Moratilla C, Cejas P (2006). MKP1/CL100 controls tumor growth and sensitivity to cisplatin in non-small-cell lung cancer. Oncogene.

[53] Ibanez de Caceres I, Dulaimi E, Hoffman AM, Al-Saleem T, Uzzo RG, Cairns P (2006). Identification of novel target genes by an epigenetic reactivation screen of renal cancer. Cancer Res.

[54] Takai D, Jones PA (2003). The CpG island searcher: a new WWW resource. Silico Biol.

[55] Ponger L, Mouchiroud D (2002). CpGProD: identifying CpG islands associated with transcription start sites in large genomic mammalian sequences. Bioinformatics.

[56] Takai D, Jones PA (2002). Comprehensive analysis of CpG islands in human chromosomes 21 and 22. Proc Natl Acad Sci USA.

[57] Aryee MJ, Jaffe AE, Corrada-Bravo H, Ladd-Acosta C, Feinberg AP, Hansen KD (2014). Minfi: a flexible and comprehensive Bioconductor package for the analysis of Infinium DNA methylation microarrays. Bioinformatics.

[58] Morris TJ, Butcher LM, Feber A, Teschendorff AE, Chakravarthy AR, Wojdacz TK (2014). ChAMP: 450k chip analysis methylation pipeline. Bioinformatics.

[59] Weisenberger DJ, Van Den Berg D, Pan F, Berman BP, Laird PW. Comprehensive DNA methylation analysis on the Illumina Infinium assay platform [electronic]. Illumina, San Diego; 2008 [cited 2019 03/03/2019]. Available from: https://www.semanticscholar.org/paper/Comprehensive-DNA-Methylation-Analysis-on-the-%C2%AE-%C2%AE-Weisenberger-Berg/b35c3020c913dfc555dc6eebfeeae9cd9973f5f4?citationIntent=methodology#citing-papers.

[60] Akalin A, Kormaksson M, Li S, Garrett-Bakelman FE, Figueroa ME, Melnick A (2012). methylKit: a comprehensive R package for the analysis of genome-wide DNA methylation profiles. Genome Biol.

[61] Smyth GK (2005). Limma: linear models for microarray data. Bioinformatics and computational biology solutions using R and Bioconductor.

[62] Martin M (2011). Cutadapt removes adapter sequences from high-throughput sequencing reads. EMBnet J.

[63] Li B, Dewey CN (2011). RSEM: accurate transcript quantification from RNA-Seq data with or without a reference genome. BMC Bioinform.

[64] McCarthy DJ, Chen Y, Smyth GK. Differential expression analysis of multifactor RNA-Seq experiments with respect to biological variation. Nucleic Acids Res 2012:gks042. 10.1093/nar/gks042 PMC3378882 22287627

[65] Robinson MD, Oshlack A (2010). A scaling normalization method for differential expression analysis of RNA-seq data. Genome Biol.

