# Supplementary figures and images for: Transcriptional epigenetic regulation of Fkbp1/Pax9 genes is associated with impaired sensitivity to platinum treatment in ovarian cancer
Source: Clin Epigenetics. 2021 Aug 28;13:167. doi: 10.1186/s13148-021-01149-8 (PMC8401184; doi:10.1186/s13148-021-01149-8)

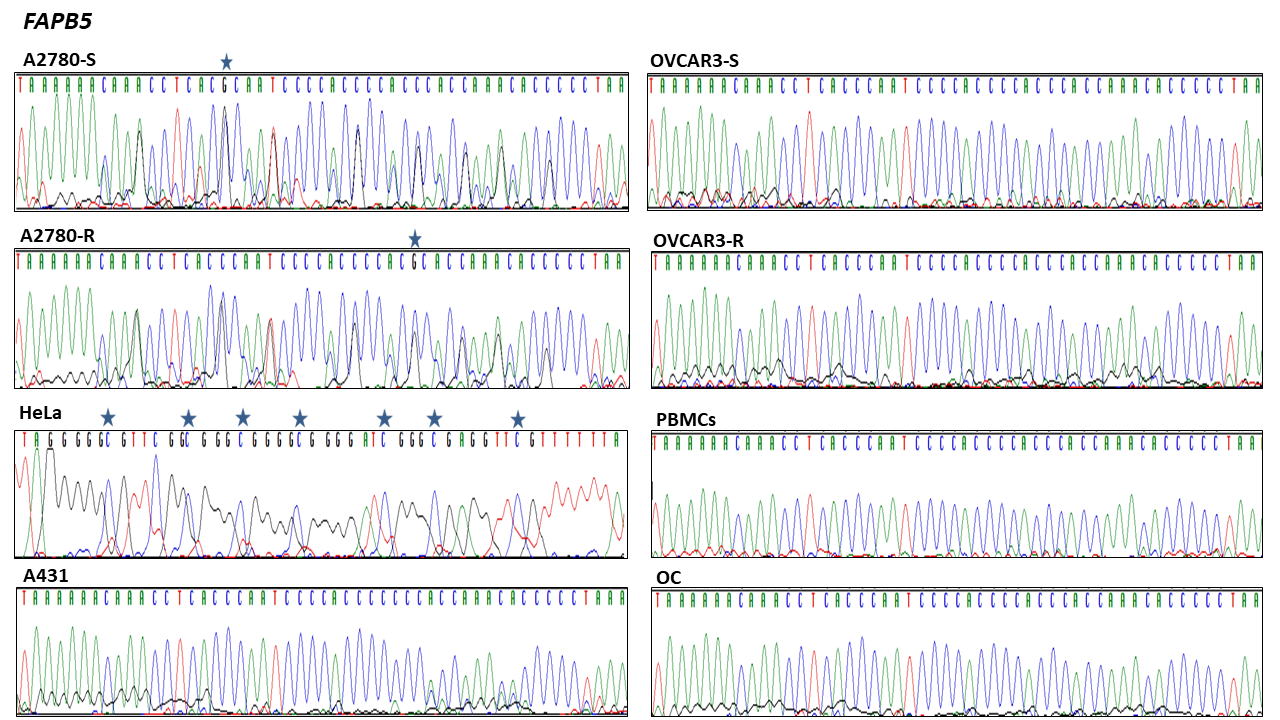

Supplement: Supplementary file 1 — Additional file 1. Supplementary Figure 1. Bisulfite sequencing of FABP5 gene. Representation of a sequence fragment from the FAPB5 gene area of bisulfite-modified DNA from sensitive and resistant A2780 and OVCAR3 tumor lines, DNA from normal ovarian tissue from patients undergoing sex change, and DNA from Peripheral Blood Mononuclear Cells (PBMCs) and tumor lines A431 and HeLa. All samples, except Hela, were sequenced with the antisense primer. Methylated positions are indicated with a blue Asterisk. [file 13148_2021_1149_MOESM1_ESM.png]

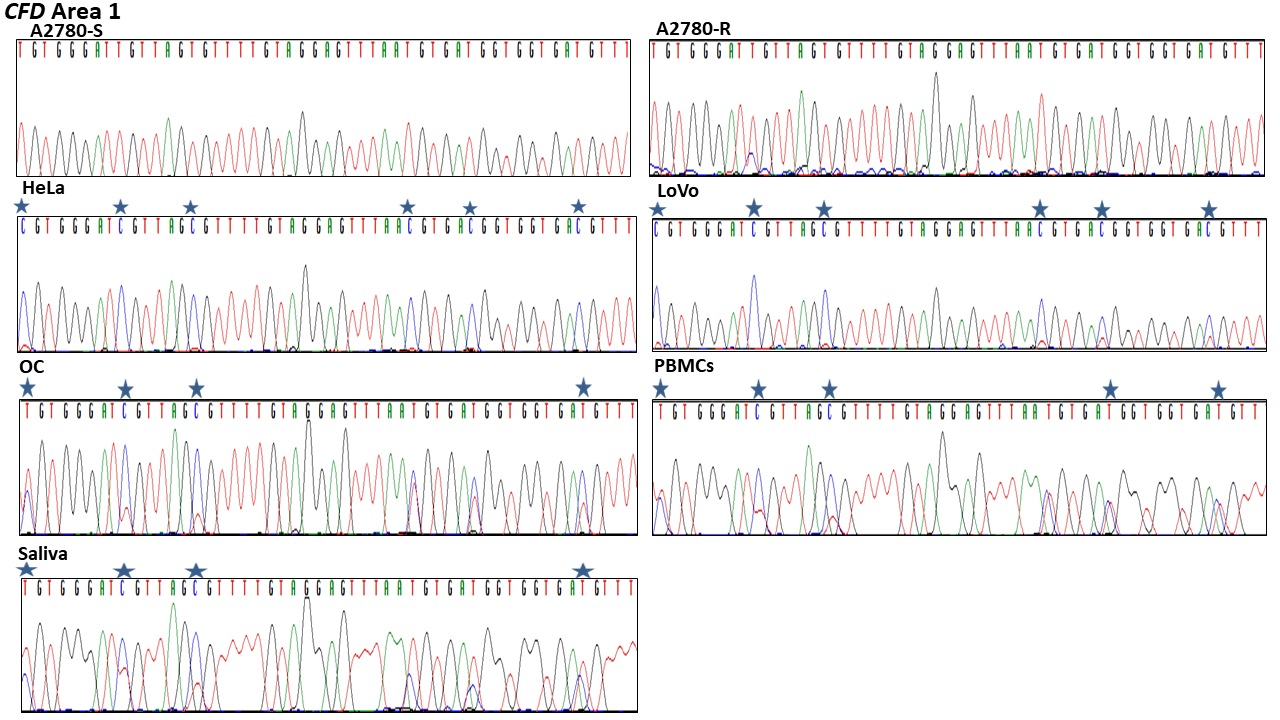

Supplement: Supplementary file 2 — Additional file 2. Supplementary Figure 2. Bisulfite sequencing of the CFD gene Area1. Representation of a sequence fragment of CFD gene of bisulfite-modified DNA from sensitive and resistant tumor lines A2780, DNA normal ovarian tissue from patients undergoing sex change and from Peripheral Blood Mononuclear Cells (PBMC's) as well DNA extracted from oral epithelium. The sequenced tumor lines were cervical cancer (HeLa) and adenocarcinoma of the colon (LoVo). All the samples were sequenced with the sense primer. Methylated positions are indicated by a blue asterisk. [file 13148_2021_1149_MOESM2_ESM.png]

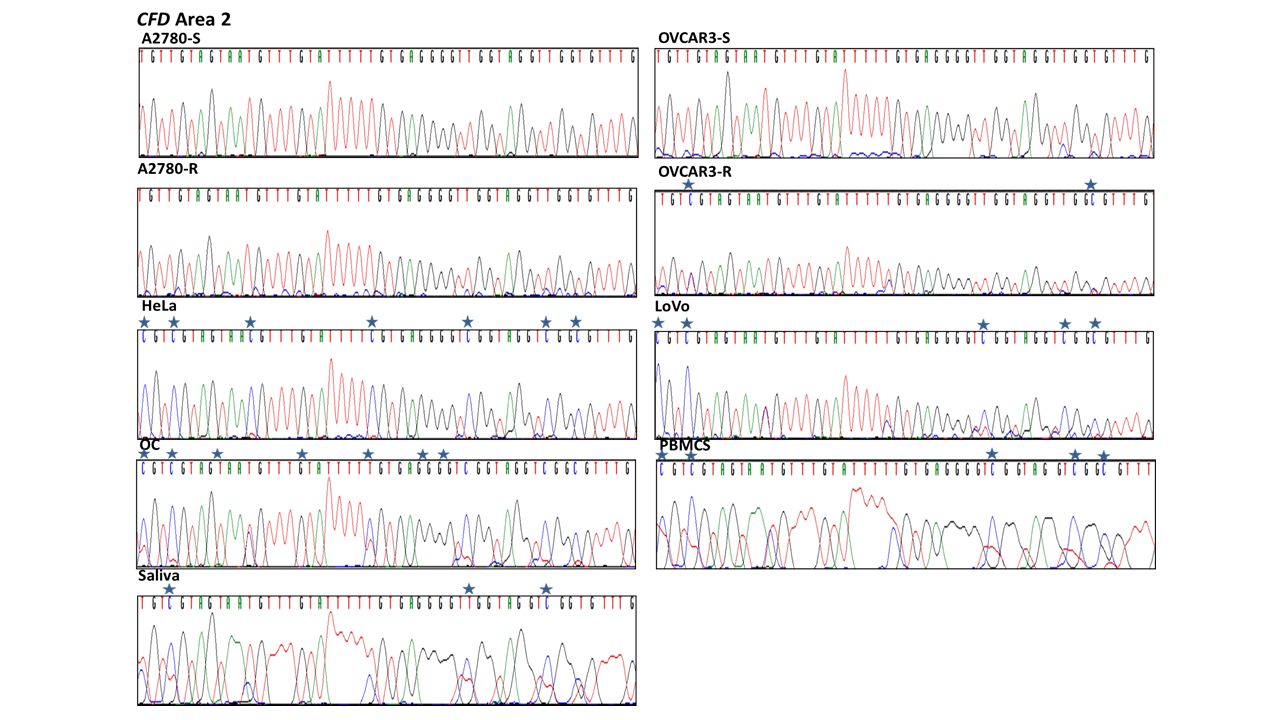

Supplement: Supplementary file 3 — Additional file 3. Supplementary Figure 3. Bisulfite sequencing of the CFD gene Area 2. Representation of a sequence fragment of the CFD gene of bisulfite-modified DNA from the sensitive and resistant A2780/ OVCAR-3 tumor lines, normal ovarian tissue from patients undergoing sex change, DNA from Peripheral Blood Mononuclear Cells (PBMCs) and DNA extracted from oral epithelium The sequenced tumor lines were cervical cancer (HeLa) and adenocarcinoma of the colon (LoVo). All the samples were sequenced with the reverse primer. Methylated positions are indicated by a blue asterisk. [file 13148_2021_1149_MOESM3_ESM.png]

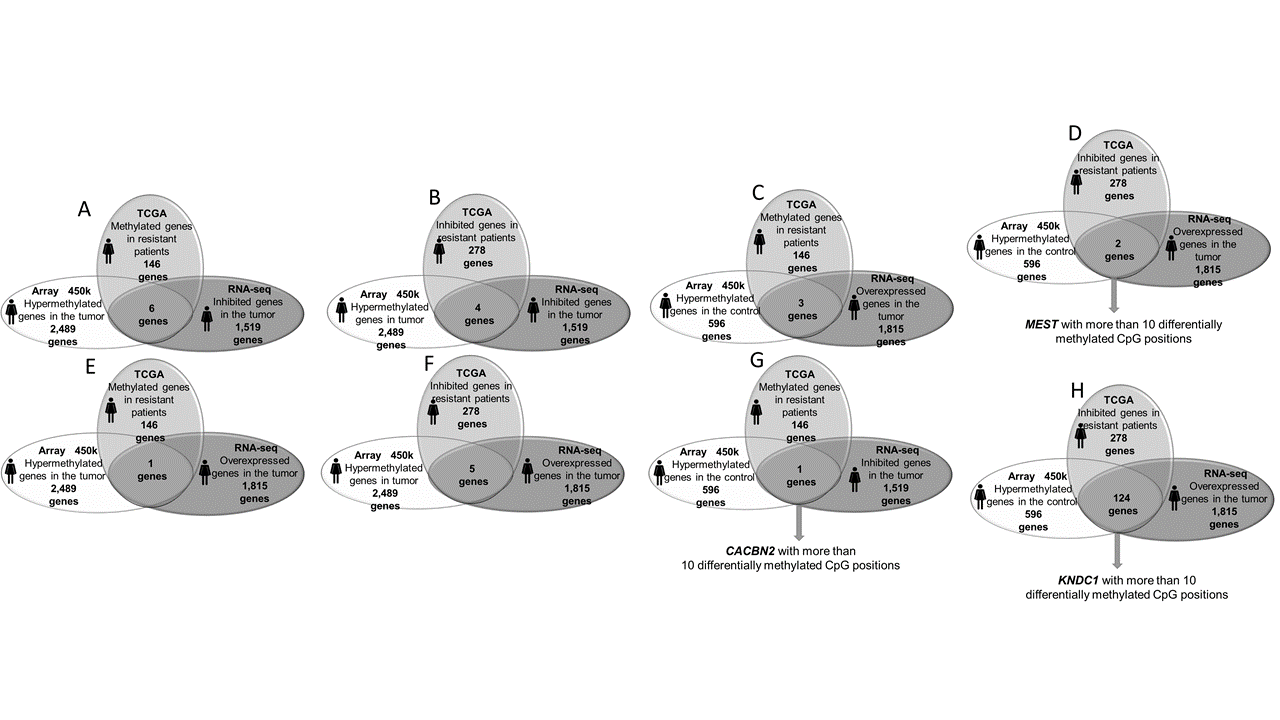

Supplement: Supplementary file 4 — Additional file 4. Supplementary Figure 4. Set of cross-analysis called group 2 designed to identify genes of interest. Venn’s diagrams A, B, C, D, E, F, G and H show genes derived from the analyzes developed between the Illumina 450K methylation array and transcriptome data obtained through RNA-seq performed on patient samples with the methylation and expression data of TCGA patients. It should be mentioned that in order not to omit possible candidates, the search for markers also included overexpressed and hypomethylated genes in the tumors of patients, since genes that resemble the profile sought in in vitro resistance could be found within such a group. Candidates exhibiting lower expression in R regarding another group of genes in S may correlate with hypomethylation in S (lower β values) or hypermethylation in R. [file 13148_2021_1149_MOESM4_ESM.png]

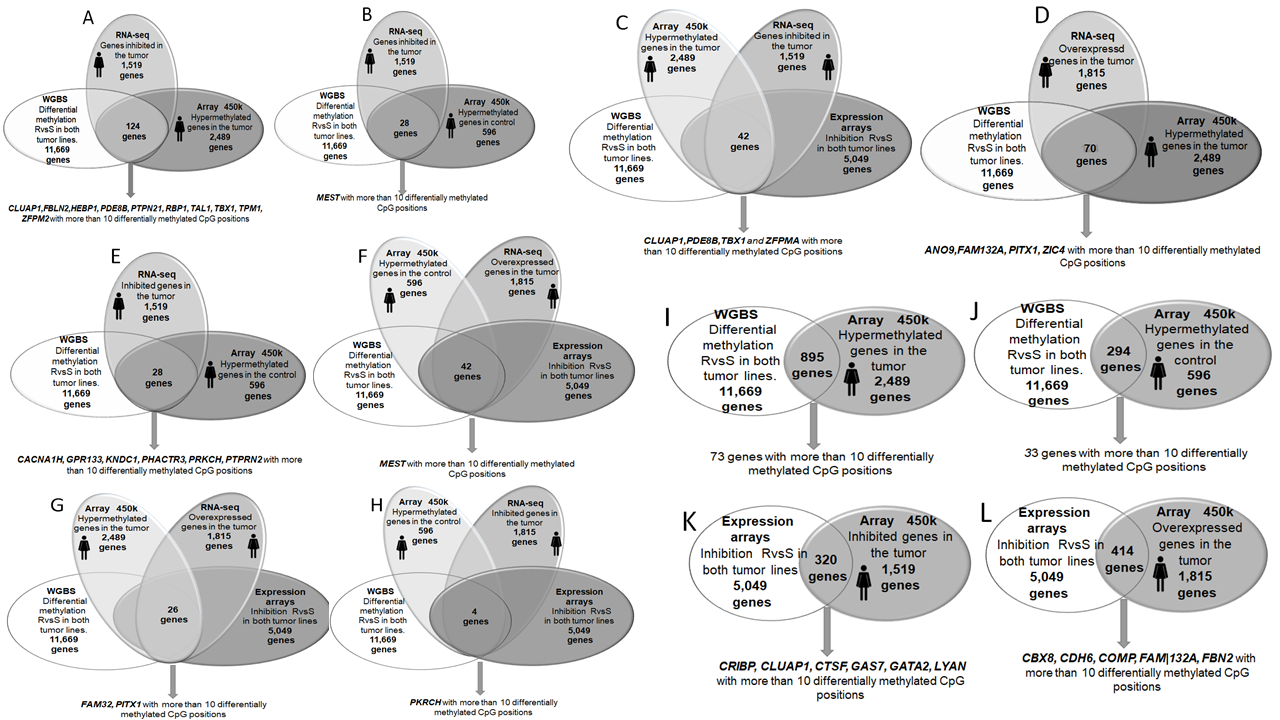

Supplement: Supplementary file 5 — Additional file 5. Supplementary Figure 5. Set of cross-analysis called group 3 designed to identify genes of interest. Venn’s diagrams A, B, C, D, E, F, G, H, I, J, K and L show genes derived from the analyzes developed between data obtained from Illumina methylation array 450K, transcriptome data obtained through RNA-seq both performed on patient samples, with methylation and expression data from the experimental model, that is, expression array and WGBS performed in vitro. It should be mentioned that in order not to omit possible candidates, the search for markers also included overexpressed and hypomethylated genes in the tumors of patients, since genes that resemble the profile sought in in vitro resistance could be found within such a group. Candidates exhibiting lower expression in R regarding another group of genes in S may correlate with hypomethylation in S (lower β values) or hypermethylation in R. [file 13148_2021_1149_MOESM5_ESM.png]

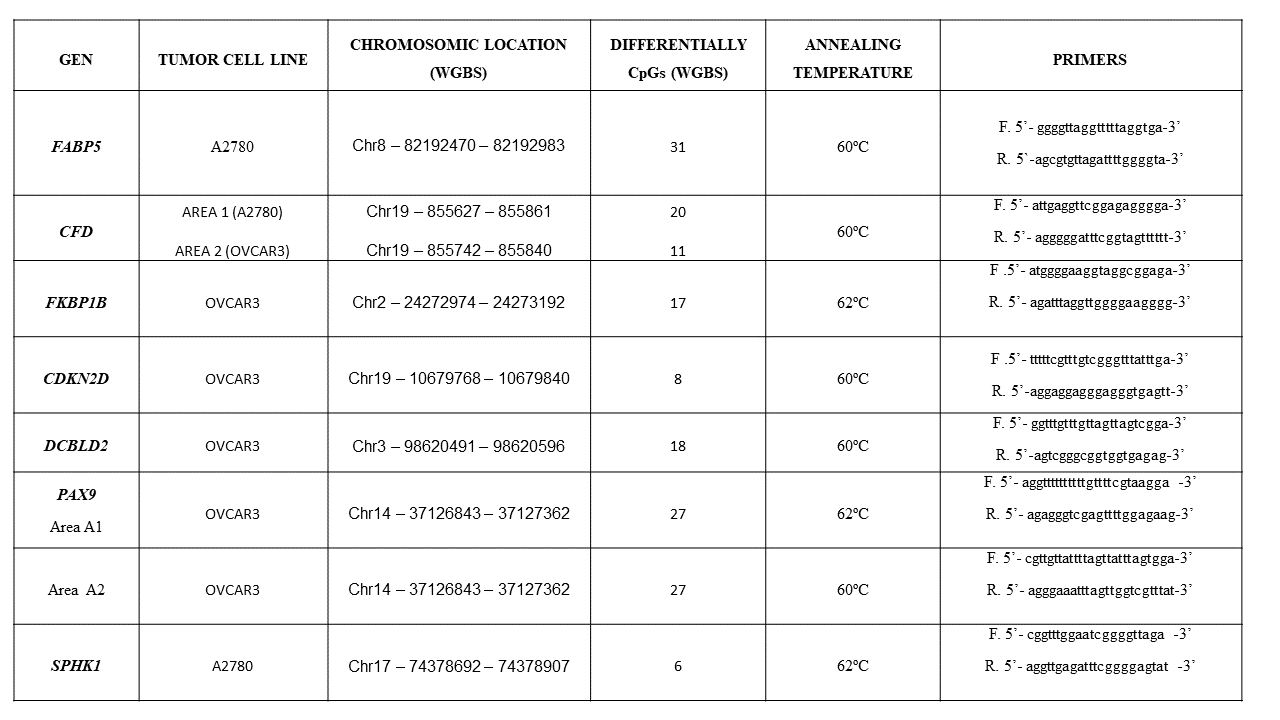

Supplement: Supplementary file 6 — Additional file 6. Supplementary Table 1. Bisulfite PCR amplification features of initial candidate genes. Genes with differentially methylated regions obtained by WGBS in the OVCAR3 (S/R) and A2780 (S/R) lines were validated by bisulfite sequencing and further analyzed in additional tumor lines, in order to know the methylation frequency of those CpG positions. This analysis allowed the subsequent design of specific oligonucleotides for methylated and unmethylated positions in methylation-specific PCR. Here it is also shown the chromosomal location of the region observed as differentially methylated by methylome sequencing, the cell line in which that region was identified and the number of CpG (CG dinucleotides) in which the methylation mark was found when resistance vs. sensitivity was contrasted. The amplification conditions for PCR were 5' at 95º, 40 cycles (1' at 95ºC, 1' at 60 or 62º [Annealing temperature for each gene was obtained by performing a gradient PCR], 1' at 72º and a final extension of 8' at 72ºC. PAX9 region was splitted into two areas due to its length. F: forward sense, R: reverse sense. [file 13148_2021_1149_MOESM6_ESM.png]

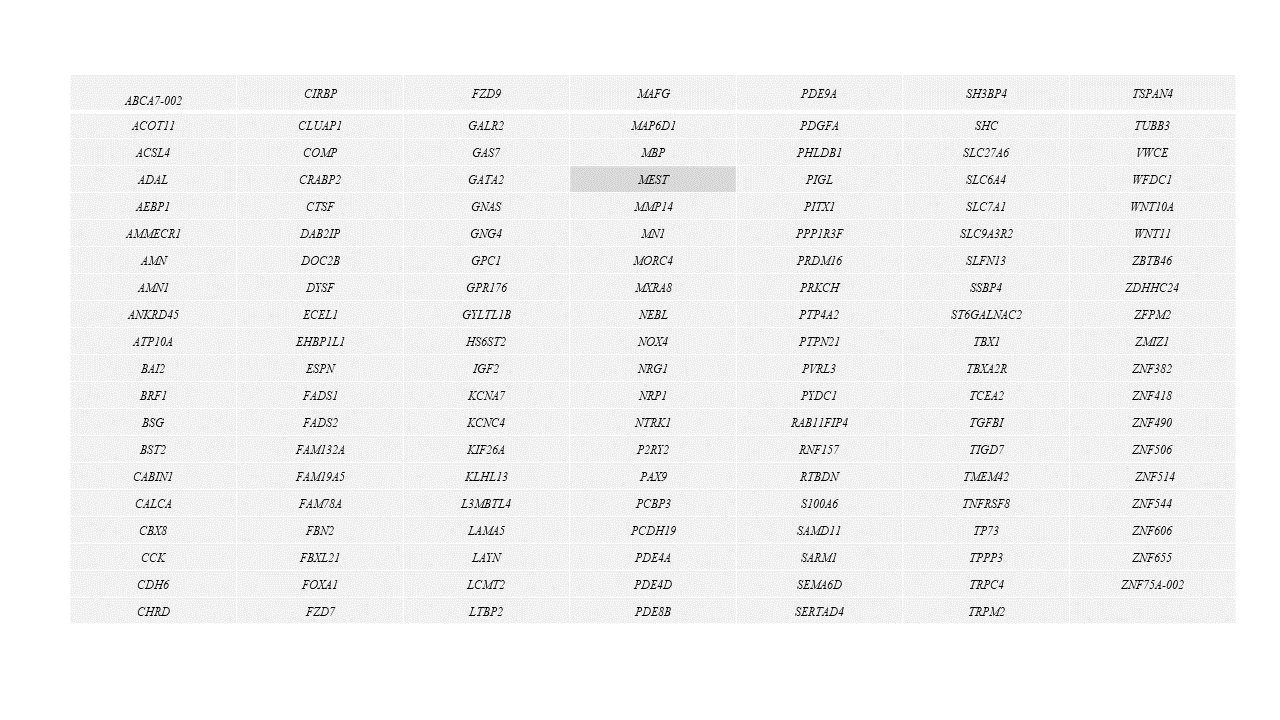

Supplement: Supplementary file 7 — Additional file 7. Supplementary Table 2. Genes derived from analysis of contrast B Group 1 (Figure 6) with more than 10 positions differentially CpG methylated. MEST gen is highlighted. [file 13148_2021_1149_MOESM7_ESM.png]

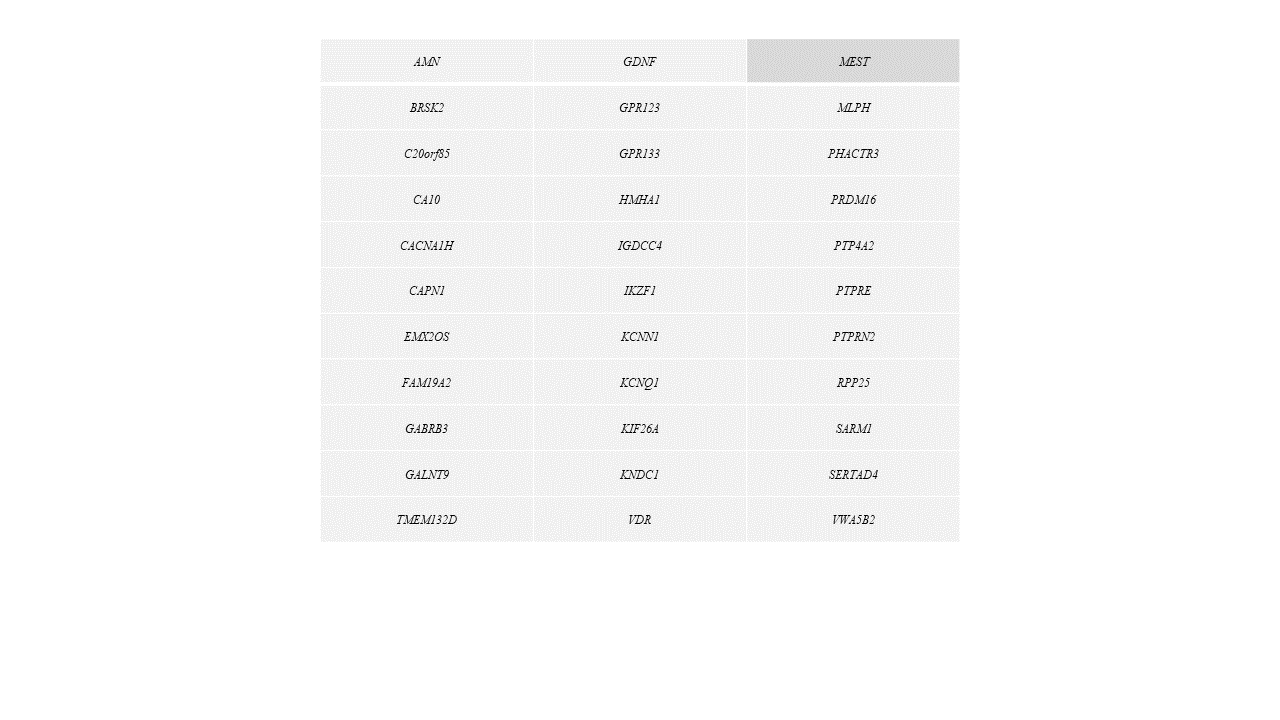

Supplement: Supplementary file 8 — Additional file 8. Supplementary Table 3. Genes resulting from contrast J Group 3 (Figure S5) with more than 10 positions differentially CpG methylated. MEST gen is highlighted [file 13148_2021_1149_MOESM8_ESM.png]

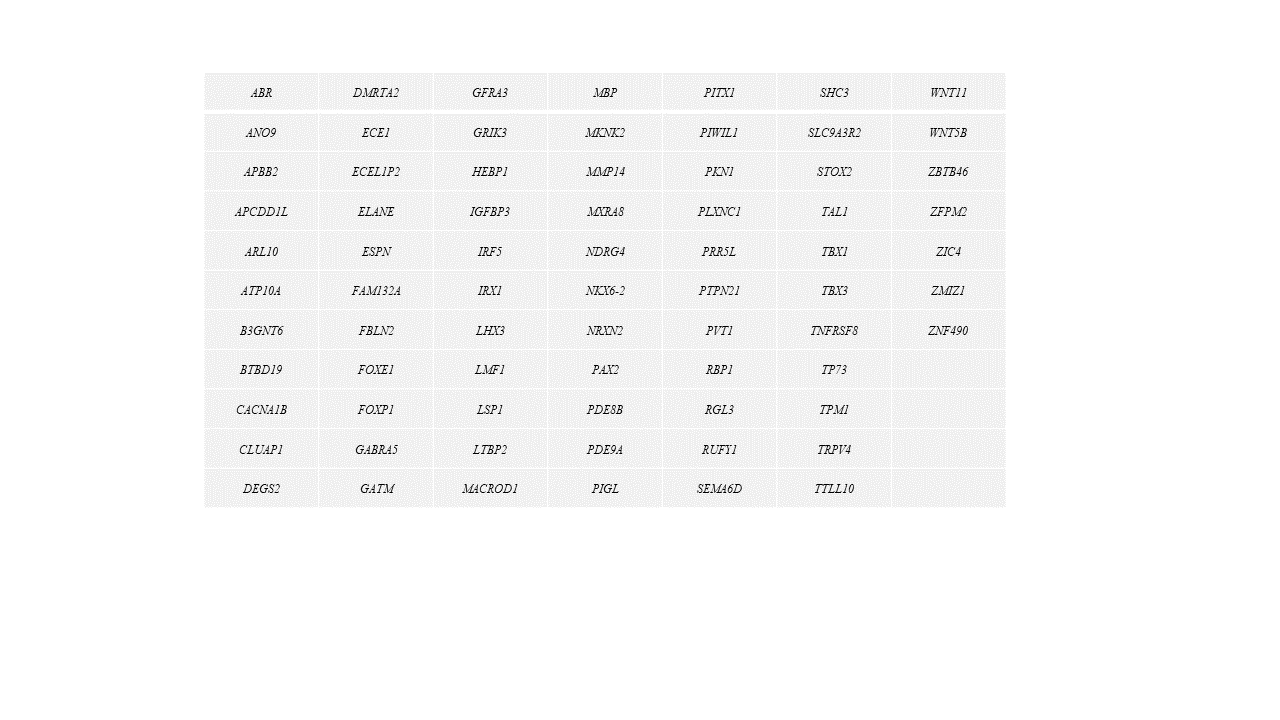

Supplement: Supplementary file 9 — Additional file 9. Supplementary Table 4. Genes resulting from contrast I Group 3 (Figure S5) with more than 10 positions differentially CpG methylated. [file 13148_2021_1149_MOESM9_ESM.png]

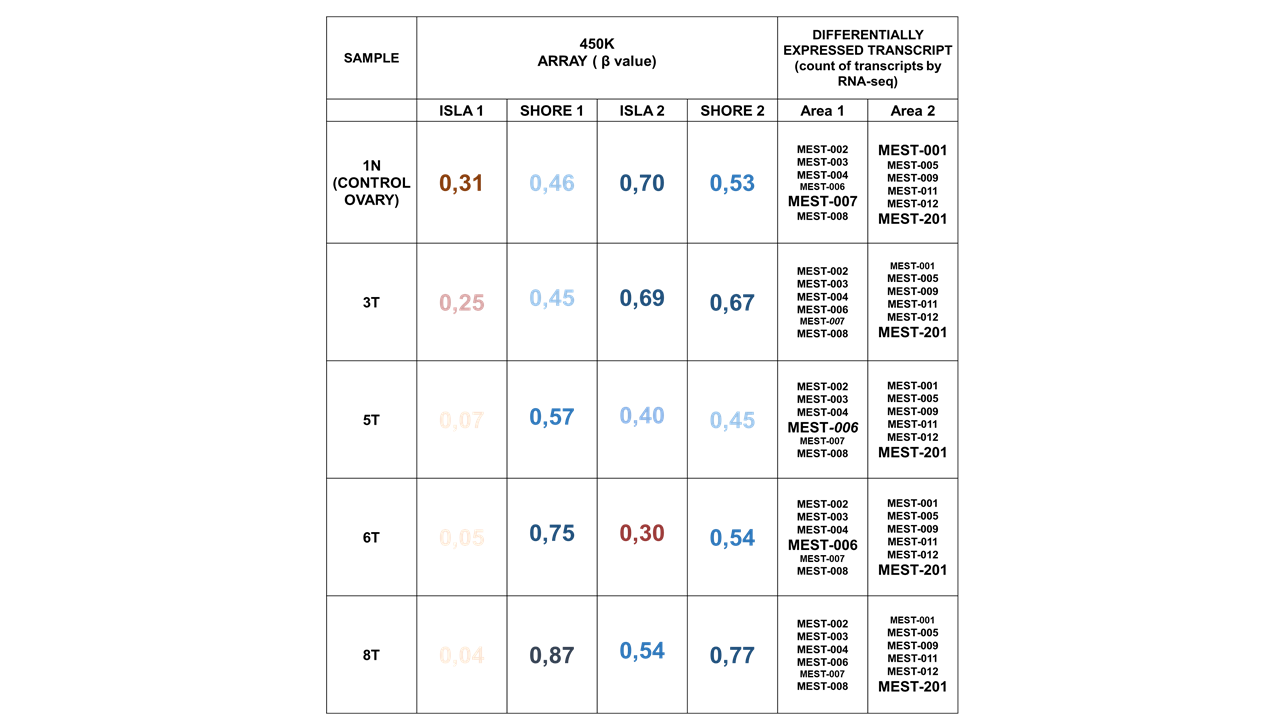

Supplement: Supplementary file 10 — Additional file 10. Supplementary Table 5. Methylation and expression cross-analysis regarding the CpG islands and shores of MEST gen. Based on the data obtained from RNA-seq and the Illumina 450K array performed in our patients, we did a cross-analysis between methylation and expression of each of these islands and their associated shore regions, with the aim of identifying the influence that the degree of methylation of these areas may exert on the regulation of the expression of MEST transcripts patients. The level of methylation in the samples was assessed by the study of the β value using the same range as that used in the screening of potential genes in the first approach. Blue color represents hypermethylation and the red color hypomethylation. The inhibited transcripts are represented in smaller size and those over-expressed in larger. [file 13148_2021_1149_MOESM10_ESM.png]

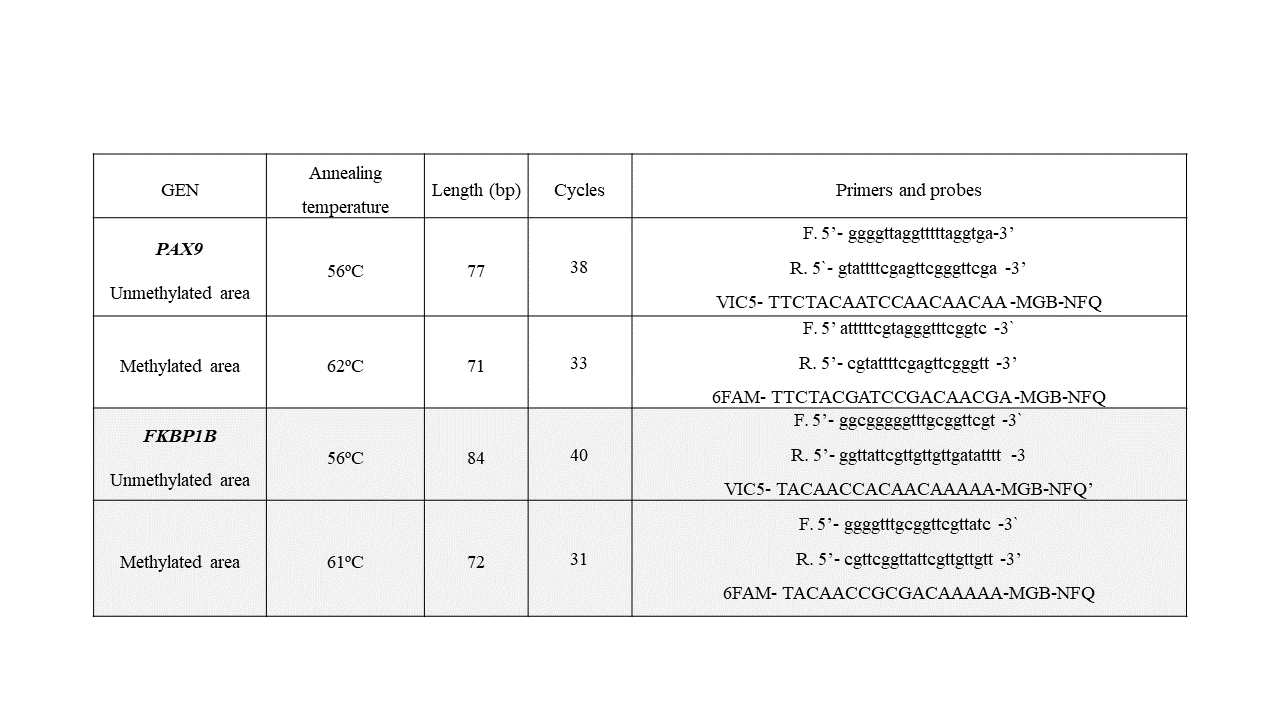

Supplement: Supplementary file 11 — Additional file 11. Supplementary Table 6. Specific methylation amplification features of PAX9 and FKBP1B genes. Once the methylation frequencies of the different CpG positions in the tumor lines were analyzed through bisulfite sequencing, those with the highest were chosen to perform the Methylation Specific PCR technique in the different cohorts of ovarian cancer patients. PCR reactions were performed on primary tumors and control samples and amplification conditions depended on the gradient reactions performed for each of the genes, varying in both cycles and annealing temperatures. The amplification conditions for PCR were 5' at 95º, 8' at 50ºC, the number of cycles depended on each gene (1' to 95ºC, annealing temperature for each gene was obtained by performing a temperature gradient PCR. Annealing was a 1’ long and extension for 1' at 72ºC) and a final extension of 8' at 72ºC. Primers and probes used to amplify the methylated and unmethylated areas of each gene of interest are also shown. Probes are labeled with fluorophores for the quantitative determination of methylation in these genes through quantitative MSP (qMSP). F: forward sense primer, R: reverse sense primer. [file 13148_2021_1149_MOESM11_ESM.png]
