# Supplementary material for: Transcriptional epigenetic regulation of Fkbp1/Pax9 genes is associated with impaired sensitivity to platinum treatment in ovarian cancer
Source: Clin Epigenetics. 2021 Aug 28;13:167. doi: 10.1186/s13148-021-01149-8 (PMC8401184; doi:10.1186/s13148-021-01149-8)
Supplement: Supplementary file 12 — Additional file 12. Word document containing supplementary information related to the protocols of the techniques used in the development of this study. [file 13148_2021_1149_MOESM12_ESM.docx]

Supplementary Materials and Methods

Transcriptional epigenetic regulation of Fkbp1/Pax9 genes is associated with impaired sensitivity to platinum treatment in ovarian cancer.

*Gene expression assays.*

Total RNA from sensitive (S), resistant (R) and resistant under epigenetic reactivation treatment (RT) cells was extracted by the guanidine thiocyanate method using TRIZOL reagent (Invitrogen, CA) and purified with the miRNeasy mini kit (Quiagen, CA), combined with DNAsa treatment as recommended by Agilent. RNA Integrity was determined by running samples in a 2100 Bioanalyzer (Agilent Technologies).

**Gene expression microarrays:** To amplify and label the RNA, the protocol of Gene Expression Analysis based on single-color microarray (Agilent Technologies, USA) was followed. 1 μg of total RNA was retrotranscribed using the T7 promoter primer and the MMLV retrotranscriptase. Subsequently, the cDNA was converted to an aRNA (amplified) using the T7 RNA polymerase which simultaneously amplifies and incorporates the labeled fluorophore marked with cyanine 3 (Cy3).

Later, samples were hybridized in the "Complete Human Genome Microarray" 4 x 44 K (G4112F, Agilent Technologies, USA) with biological duplicates for each experimental group. 1.65 μg of cyc-labeled aRNA (amplified RNA) was incubated for 17 hours in a 65 ° hybridization oven (G2545A, Agilent) at a final concentration of 1X in the HI-RPM hybridization buffer as recommended by the manufacturer’s instructions. The arrays were then washed according to commercial recommendations and dried by centrifugation. The scanning was performed at a resolution of 5 μm and the images were analyzed through the "Feature extraction software". Output data was normalized through the VSN (Normalization of Variance Stabilization) method [1] which preserves the biological characteristics of the data and stabilizes the variance throughout the intensity range. Once the data was normalized, three types of filters were applied, expression, label and error.

**qPCR:** Real-time PCR methodology are based on two main steps. The first one consisted in the retro transcription of total RNA, starting from a concentration of 500 ng / μl for tumors and 1000 ng / ul for cell lines. Takara kit (PrimeScriptTM RT reagent Perfect Real Time ID Kit: # RR037A Condalab, Spain) was used to obtain the cDNA. The second step was the valuation of the cDNA through specific Taqman probes (Life Technologies, Spain) and the use of the 5x HOT Mix Master FIREPol® Probe qPCR Mix Plus (ROX) (Genycell Biotech, Spain). In each reaction, 1.6 μl of cDNA was used in a final volume of 10 μl following the parameters established by the manufacturer’s instructions. The amplification conditions were: 15 'at 95°C and 40 cycles (15 "at 95°C, 1' at 60°C).

**RNA-seq:** RNA extraction of samples embedded in OCT (Optimal Cutting Temperature compound) was performed by the same methodology explained in previous sections. In the case of the fallopian ductal samples, the extraction required a previous mechanical treatment for tissue homogenization. Total RNA belonging to a normal ovarian sample (tubal ligation) and 7 tumor samples was sent to the National Center for Cardiovascular Research Foundation (CNIC) at a concentration of 1 μg of RNA in 100 μl of DEPC water. Sequencing was performed through coupled-end libraries in a Illumina Hiseq 2500 sequencer. In order to expand the number of analyzed samples, 5 normal samples of fallopian ductal tissue and 5 tumor samples (1 μg) were sent to Sistemas Genómicos (Valencia). The protocol followed in Genomic Systems was as follows: between 100-200 ng of RNA were used and its quality and quantity was determined in Bioanlayzer 2100 and Qubit 2.0. The poly (A) + mRNA fraction was isolated from the total RNA and the cDNA libraries were obtained following the recommendations of Illumina. The poly (A) + RNA was isolated by magnetic beads bound to oligo-poly T and was chemically fragmented before retro-transcription. The cDNA fragments were subjected to a final repair process through the addition of a single "A" base to the 3 'end and then the ligation of the adapters was performed. Finally, the products were purified and enriched by PCR to create the final double-stranded cDNA library indexed. The quality of the libraries was analyzed in Bioanalyzer 2100 through the High Sensitivity assay. The amount of the libraries was determined by real-time PCR in LightCycler 480 (Roche). The library set was subjected to paired end sequencing (100 x 2) in the Illumina HiSeq2500. 18 samples were sequenced (6 controls and 12 tumors).

*Epigenetic validation.*

**DNA extraction**.

Procedures described below refer to the DNA extraction from samples of different sources based on the phenol / chloroform protocol, which is based on the organic and inorganic phases separation [2]

* Cell lines extraction: 1 ml of PBS was added to frozen cells and then scraped to detached them from the plate. The extract was collected and centrifuged for 5 minutes at 14,000 RPM at 4°C. In the case that cells were not frozen they were trypsinized and centrifuged and the cellular precipitate was collected for the subsequent extraction. The cellular precipitate of the two types of samples was digested by adding 200 μl of PBS 1x + 2mM EDTA, 350 μl of DNA B (5M NaCl + 0.5 M EDTA), 40 μl of SDS and 10 μl of proteinase K (PK 10). ug / ml) (Quiagen, USA). The temperature at which the digestion was carried out was 50°C for two hours and then at 37°C overnight. The next day the DNA extraction was performed by the phenol / chloroform method (Merck, USA).

* Fresh tissue extraction: Fresh samples embedded in OCT comprised 10 cuts of 10 μm thick. 1 ml of PBS (free of Ca-Mg) (Biochrom, UK) was added to each sample to dissolve the OCT. The mix was centrifuged for 5 minutes at 14,000 RPM and the supernatant was carefully removed. Washing and centrifugation with PBS was repeated and digestion and extraction were carried out as described before. For the extraction from Fallopian tubes, these were previously homogenized by the Power Biomasher II gun and proceeded to the extraction as described in the previous section.

* Extraction from paraffin blocks (FFEP): Each sample comprised 10 cuts of 10 μm thick. Deparaffinization was performed by adding 1ml of Xylene (Panreac, Barcelona, ​​Spain) to the sample and incubating for 5 minutes at 42°C. Subsequently, it was centrifuged for 5 minutes at 14,000 RPM and the supernatant was discarded. This procedure was repeated, and 1 ml of pure ethanol was added to the precipitate the sample. (Merck, USA). The mixture was centrifuged for 5 minutes at 14,000 RPM and the ethanol was removed. Once the sample was completely dry, it was digested and extracted as described before.

**Whole genome bisulfite sequencing.**

DNA from the experimental groups OVCAR3 (S / R) and A2780 (S / R) was sent to the National Center for Genomic Analysis (CNAG) in Barcelona for the analysis of complete genome methylation.

Briefly, 2μg of genomic DNA was mixed with unmethylated phage λ DNA at a rate of 5 ng per 1 μg of genomic DNA. The library was prepared using the TruSeq ™ DNA sample preparation kit v2 (Illumina Inc) following manufacturer’s instructions with minimal modifications. The DNA was sonicated using a Covaris E220 (Covaris Inc, USA) to generate fragments between 50-500 bp. The selected size for the elaboration of libraries was of 150-300 pb. Subsequently, the fragments were purified through AMPure XP spheres (Agencourt Bioscience Corp), repaired, adenylated and linked to specific paired ends adapters (Illumina Inc). After ligation, the fragments were sodium bisulfite modified using the EpiTect Bisulfite kit (Qiagen, USA) following the indications. Modified DNA and linked to the adapters was amplified in 7 PCR cycles using the PfuTurboCx Hotstart DNA polymerase (Agilent Technologies, USA). The quality control of the library was carried out through an assay in the BioAnalyzer 7500 (Agilent Technologies, USA).

**REFERENCES**.

1. Huber, W., et al., *Variance stabilization applied to microarray data calibration and to the quantification of differential expression.* Bioinformatics, 2002. **18**(suppl_1): p. S96-S104.

2. Sambrook, J., E.F. Fritsch, and T. Maniatis, *Molecular cloning: a laboratory manual*. 1989: Cold spring harbor laboratory press.

Supplementary Table 1. Bisulfite PCR amplification features of initial candidate genes. Genes with differentially methylated regions obtained by WGBS in the OVCAR3 (S/R) and A2780 (S/R) lines were validated by bisulfite sequencing and further analyzed in additional tumor lines, in order to know the methylation frequency of those CpG positions. This analysis allowed the subsequent design of specific oligonucleotides for methylated and unmethylated positions in methylation-specific PCR. Here it is also shown the chromosomal location of the region observed as differentially methylated by methylome sequencing, the cell line in which that region was identified and the number of CpG (CG dinucleotides) in which the methylation mark was found when resistance vs. sensitivity was contrasted. The amplification conditions for PCR were 5' at 95º, 40 cycles (1' at 95ºC, 1' at 60 or 62º [Annealing temperature for each gene was obtained by performing a gradient PCR], 1' at 72º and a final extension of 8' at 72ºC. *PAX9* region was splitted into two areas due to its length. F: forward sense, R: reverse sense.

Supplementary Figure 1. Bisulfite sequencing of *FABP5* gene. Representation of a sequence fragment from the *FAPB5* gene area of bisulfite-modified DNA from sensitive and resistant A2780 and OVCAR3 tumor lines, DNA from normal ovarian tissue from patients undergoing sex change~~,~~ and ~~DNA~~ from Peripheral Blood Mononuclear Cells (PBMCs) and tumor lines A431 and HeLa. All samples, except Hela, were sequenced with the antisense primer. Methylated positions are indicated with a blue Asterisk.

Supplementary Figure 2. Bisulfite sequencing of the *CFD* gene Area1. Representation of a sequence fragment of *CFD* gene of bisulfite-modified DNA from sensitive and resistant tumor lines A2780, DNA normal ovarian tissue from patients undergoing sex change and from Peripheral Blood Mononuclear Cells (PBMC's) as well DNA extracted from oral epithelium. The sequenced tumor lines were cervical cancer (HeLa) and adenocarcinoma of the colon (LoVo). All the samples were sequenced with the sense primer. Methylated positions are indicated by a blue asterisk

Supplementary Figure 3. Bisulfite sequencing of the *CFD* gene Area 2. Representation of a sequence fragment of the *CFD* gene of bisulfite-modified DNA from the sensitive and resistant A2780/ OVCAR-3 tumor lines, normal ovarian tissue from patients undergoing sex change, DNA from Peripheral Blood Mononuclear Cells (PBMCs) and DNA extracted from oral epithelium The sequenced tumor lines were cervical cancer (HeLa) and adenocarcinoma of the colon (LoVo). All the samples were sequenced with the reverse primer. Methylated positions are indicated by a blue asterisk.

Supplementary Table 2. Genes derived from analysis of contrast B Group 1 (Figure 6) with more than 10 positions differentially CpG methylated. *MEST* gen is highlighted.

Supplementary Figure 4. Set of cross-analysis called group 2 designed to identify genes of interest. Venn’s diagrams A, B, C, D, E, F, G and H show genes derived from the analyzes developed between the Illumina 450K methylation array and transcriptome data obtained through RNA-seq performed on patient samples with the methylation and expression data of TCGA patients. It should be mentioned that in order not to omit possible candidates, the search for markers also included overexpressed and hypomethylated genes in the tumors of patients, since genes that resemble the profile sought in in vitro resistance could be found within such a group. Candidates exhibiting lower expression in R regarding another group of genes in S may correlate with hypomethylation in S (lower β values) or hypermethylation in R.

Supplementary Figure 5. Set of cross-analysis called group 3 designed to identify genes of interest. Venn’s diagrams A, B, C, D, E, F, G, H, I, J, K and L show genes derived from the analyzes developed between data obtained from Illumina methylation array 450K, transcriptome data obtained through RNA-seq both performed on patient samples, with methylation and expression data from the experimental model, that is, expression array and WGBS performed in vitro. It should be mentioned that in order not to omit possible candidates, the search for markers also included overexpressed and hypomethylated genes in the tumors of patients, since genes that resemble the profile sought in in vitro resistance could be found within such a group. Candidates exhibiting lower expression in R regarding another group of genes in S may correlate with hypomethylation in S (lower β values) or hypermethylation in R.

Supplementary Table 3. Genes resulting from contrast J Group 3 (Figure S5) with more than 10 positions differentially CpG methylated. *MEST* gen is highlighted.

Supplementary Table 4. Genes resulting from contrast I Group 3 (Figure S5) with more than 10 positions differentially CpG methylated.

Supplementary Table 5. Methylation and expression cross-analysis regarding the CpG islands and shores of *MEST* gen. Based on the data obtained from RNA-seq and the Illumina 450K array performed in our patients, we did a cross-analysis between methylation and expression of each of these islands and their associated shore regions, with the aim of identifying the influence that the degree of methylation of these areas may exert on the regulation of the expression of MEST transcripts patients. The level of methylation in the samples was assessed by the study of the β value using the same range as that used in the screening of potential genes in the first approach. Blue color represents hypermethylation and the red color hypomethylation. The inhibited transcripts are represented in smaller size and those over-expressed in larger.

Supplementary Table 6. Specific methylation amplification features of *PAX9* and *FKBP1B* genes. Once the methylation frequencies of the different CpG positions in the tumor lines were analyzed through bisulfite sequencing, those with the highest were chosen to perform the Methylation Specific PCR technique in the different cohorts of ovarian cancer patients. PCR reactions were performed on primary tumors and control samples and amplification conditions depended on the gradient reactions performed for each of the genes, varying in both cycles and annealing temperatures. The amplification conditions for PCR were 5' at 95º, 8' at 50ºC, the number of cycles depended on each gene (1' to 95ºC, annealing temperature for each gene was obtained by performing a temperature gradient PCR. Annealing was a 1’ long and extension for 1' at 72ºC) and a final extension of 8' at 72ºC. Primers and probes used to amplify the methylated and unmethylated areas of each gene of interest are also shown. Probes are labeled with fluorophores for the quantitative determination of methylation in these genes through quantitative MSP (qMSP). F: forward sense primer, R: reverse sense primer.
